# Population sequencing of two endocannabinoid metabolic genes identifies rare and common regulatory variants associated with extreme obesity and metabolite level

**DOI:** 10.1186/gb-2010-11-11-r118

**Published:** 2010-11-30

**Authors:** Olivier Harismendy, Vikas Bansal, Gaurav Bhatia, Masakazu Nakano, Michael Scott, Xiaoyun Wang, Colette Dib, Edouard Turlotte, Jack C Sipe, Sarah S Murray, Jean Francois Deleuze, Vineet Bafna, Eric J Topol, Kelly A Frazer

**Affiliations:** 1 Moores UCSD Cancer Center, University of California San Diego, 9500 Gilman Drive, La Jolla, CA 92093, USA; 2 Department of Pediatrics and Rady's Childrens Hospital, University of California San Diego, 9500 Gilman Drive, La Jolla, CA 92093, USA; 3 Scripps Genomic Medicine, Scripps Translational Science Institute, 3344 North Torrey Pines Court Suite 300, La Jolla, CA 92037, USA; 4 Department of Computer Sciences, University of California San Diego, 9500 Gilman Drive, La Jolla, CA 92093, USA; 5 Department of Molecular and Experimental Medicine, The Scripps Research Institute, 10550 North Torrey Pines Road, La Jolla, CA 92037, USA; 6 Sanofi-Aventis Evry Genetics Center, 2 rue Gaston Cremieux, 91057 Evry, France; 7 Institute for Genomic Medicine, University of California San Diego, 9500 Gilman Drive, La Jolla, CA 92093, USA

## Abstract

**Background:**

Targeted re-sequencing of candidate genes in individuals at the extremes of a quantitative phenotype distribution is a method of choice to gain information on the contribution of rare variants to disease susceptibility. The endocannabinoid system mediates signaling in the brain and peripheral tissues involved in the regulation of energy balance, is highly active in obese patients, and represents a strong candidate pathway to examine for genetic association with body mass index (BMI).

**Results:**

We sequenced two intervals (covering 188 kb) encoding the endocannabinoid metabolic enzymes fatty-acid amide hydrolase (FAAH) and monoglyceride lipase (MGLL) in 147 normal controls and 142 extremely obese cases. After applying quality filters, we called 1,393 high quality single nucleotide variants, 55% of which are rare, and 143 indels. Using single marker tests and collapsed marker tests, we identified four intervals associated with BMI: the *FAAH *promoter, the *MGLL *promoter, *MGLL *intron 2, and *MGLL *intron 3. Two of these intervals are composed of rare variants and the majority of the associated variants are located in promoter sequences or in predicted transcriptional enhancers, suggesting a regulatory role. The set of rare variants in the FAAH promoter associated with BMI is also associated with increased level of FAAH substrate anandamide, further implicating a functional role in obesity.

**Conclusions:**

Our study, which is one of the first reports of a sequence-based association study using next-generation sequencing of candidate genes, provides insights into study design and analysis approaches and demonstrates the importance of examining regulatory elements rather than exclusively focusing on exon sequences.

## Background

During the past decade, the search for the underlying genetic basis of complex traits and diseases in humans has been focused on common DNA variants with a minor allele frequency (MAF) > 0.05. This approach is based on the common variant common disease hypothesis [1], our increased knowledge of common variants [2], and improved genotyping methods [3]. The effort of the human genetics community has led, through genome-wide association studies (GWASs), to the identification of over 400 genetic loci associated with complex traits. However, GWASs have uncovered only a small fraction of the estimated heritability underlying complex phenotypes. The missing heritability is potentially accounted for by rare variants or variants in epistasis, both of which are difficult to identify via current genome-wide genotyping and analysis strategies. It has been suggested that sequencing candidate genes relevant to diseases in subjects at the tails of the distribution of a quantitative trait will be an efficient means to examine the contribution of rare variants to the phenotype [4].

Obesity is highly heritable [5] and recent GWASs have identified variants in approximately 15 genes that are associated with body mass index (BMI), among which are *FTO *[6], *MC4R *[7] and *CTNNBL1 *[8]. However, taken together these genes explain only a small fraction of the disease heritability [5]. There is little overlap between the genes identified by GWASs and previous genes identified through linkage or candidate gene studies, suggesting that the approaches have different sensitivities, likely due to the fact that GWASs examine only common variants and require stringent multiple-testing corrections. The genes associated with obesity risk to date are involved in several processes, such as adipogenesis, energy balance, appetite and satiety regulation. Genes in the endocannabinoid (EC) system are known to also be involved in regulating physiological functions associated with obesity [9,10]; the EC receptor 1 gene, *CNR1*, has been genetically associated with the trait [11]. ECs have modulatory effects on energy homeostasis by binding to cannabinoid receptors in the central nervous system or peripheral tissues, regulating appetite, food intake or eating behaviors [12,13]. Deregulation of the EC system has been shown in overweight and eating disorders, and increased levels of ECs in many tissues is linked to obesity [14,15].

The fatty-acid amide hydrolase (*FAAH*) and the monoglyceride lipase (*MGLL*) genes encode enzymes of the EC system; these catabolize anandamide (AEA) and 2-arachidonyl glycerol (2-AG), respectively. Thus, FAAH and MGLL enzymatic activity or expression plays a primary role in regulating metabolite levels of the EC system. Circulating levels of AEA and 2-AG are higher in obese patients and *FAAH *expression level in adipose tissue is reduced [16,17]. A variant in *FAAH *(P129T) identified in obese patients results in reduced FAAH activity [18,19]. Despite this biological evidence, GWASs have not found significant association between obesity and EC system genes. Thus, *FAAH *and *MGLL *are excellent candidates to be sequenced in the extreme of the BMI distribution to find the extent of their genetic diversity and potential association of variants with obesity.

Currently, sequence-based association studies need to target specific intervals in the human genome to allow a sufficient number of samples to be examined. Several studies have examined exons to identify rare coding variants implicated in reduced sterol absorption and lower plasma levels of high-density lipoprotein [20], underlying cancer initiation and progression [21] and Mendelian diseases [22]. For complex diseases, regulatory variants affecting the expression of genes likely play an important role, thus justifying the sequencing of larger intervals, as was done for the 8q24 interval associated with colorectal cancer [23]. To the best of our knowledge, the approach of deep population sequencing of large candidate gene intervals has not yet been used for association studies. This is partly due to the fact that next-generation sequencing sample preparation and instruments are not yet optimized to sequence intervals in a large number of individuals. Additionally, the methods for using population sequence data to ascertain variant calling, including indels, are still being developed. Lastly, there is a lack of computational and experimental methods to analyze rare variants (< 1% allele frequency) associated with diseases.

In this report, we explore the genetic diversity of 188 kb of sequence encompassing the *FAAH *and *MGLL *genes in 289 individuals and use variants from the whole allelic frequency spectrum to investigate association with extreme obesity (BMI ≥40 kg/m^2^). We identify all the variants present in the two gene intervals, establish a number of quality filters to generate a set of high quality variants and perform association testing with obesity using two different approaches: a chi-square analysis appropriate for common variants (MAF > 0.01) and a collapsing method [24] for rare variants (MAF < 0.01). We identify 20 common variants in *MGLL *associated with high BMI and discover three intervals containing sets of rare variants (referred to as rare locus-variants) in both *MGLL *and *FAAH*. Most of the associated variants lie in regulatory elements, either close to the gene promoter or in transcriptional enhancers, as determined by chromatin signatures in HeLa and other cell types. In addition, we show the association of a rare locus-variant in the *FAAH *promoter with increased plasma levels of AEA, thus providing an independent validation of the genetic association with obesity.

## Results and discussion

### Selection of samples at extremes of the BMI distribution

**Figure 1:**
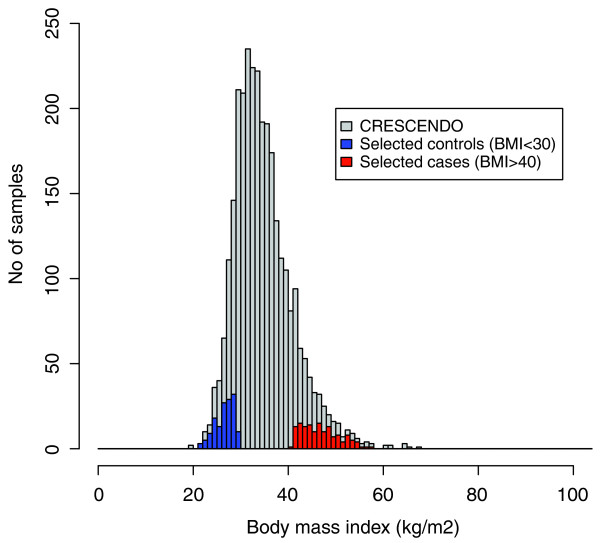
**BMI distribution in the CRESCENDO cohort (grey) and in the selected controls (147 samples with BMI ≤ 30 kg/m^2^, blue) and cases (142 samples with BMI ≥40 kg/m^2^, red)**.

To increase the power of our study to detect variants associated with extreme obesity in the *FAAH *and *MGLL *genes, we sequenced DNA from individuals at the extremes of the BMI distribution in the CRESCENDO cohort, which consists of 2,958 Caucasian individuals aged 55 years or older and was established to study obesity treatment (average BMI is 35 kg/m^2^; Figure 1). This strategy is based on the premise that a significant excess of sequence variants in one extreme compared to the other extreme that is not due to stratification is an indication of genetic association with the phenotype. We selected 289 individuals of European ancestry from both tails of the BMI distribution for both genders of the CRESCENDO cohort; 73 men and 70 women with a BMI > 40 kg/m^2 ^(referred to as cases) and 74 men and 72 women with a BMI < 30 kg/m^2 ^(referred to as controls). The cohort consists mostly of overweight people and thus only 24% of our control population has a BMI < 25 kg/m^2^. For this reason, our population is particularly well suited to identify the genetic variants associated with extreme obesity (BMI > 40 kg/m^2^).

### Targeted sequencing of 188 kb of sequence spanning *FAAH *and *MGLL*

**Figure 2:**
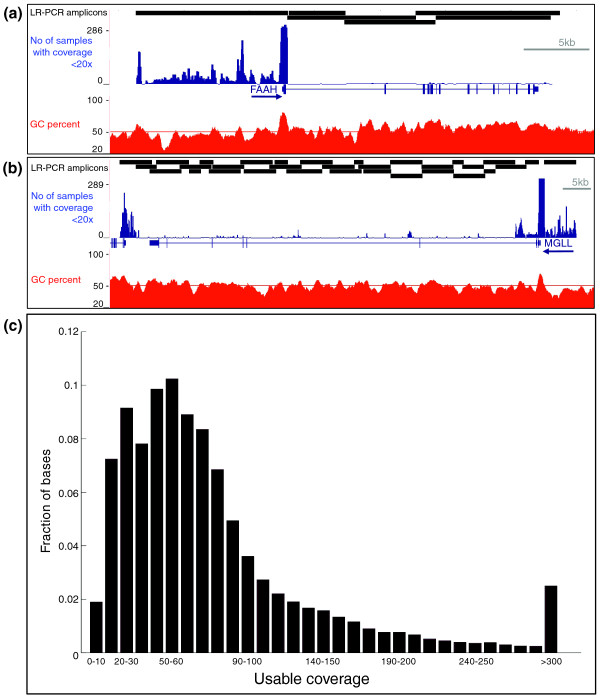
**Sequence coverage distribution**. **(a,b) **Genome Browser tracks showing locations of the 40 LR-PCR amplicons (black rectangles), the number of samples with coverage below 20× (blue histogram, 100-bp windows) and GC percent (red histogram, 10-bp windows) along the *FAAH *(a) and *MGLL *(b) re-sequenced intervals. The ends of the intervals have lower coverage due to the fact they were amplified by a single amplicon. The 5' end of the *FAAH *gene was successfully amplified but coverage is low due to difficulty sequencing high GC content regions. The high GC content at the 5' end of the *MGLL *gene resulted in an inability to successfully design PCR primer pairs despite several attempts. **(c) **Distribution of the fraction of bases (y-axis) sequenced at increasing usable coverage (x-axis) for sequence-based association studies. Usable coverage is defined at each base as the minimum coverage reached by 90% or more of the samples.

We amplified the 32-kb interval encompassing *FAAH *and the 156-kb interval encompassing *MGLL *by long range PCR (LR-PCR) using 40 overlapping amplicons (Figure 2A, B). Of the targeted base pairs, 77% were covered by two distinct amplicons and the remaining 23% (43.6 kb) located at the edges of the two intervals were covered by only one amplicon. After equimolar pooling of the amplicons, each sample was sequenced at a median coverage greater than 60× across the targeted intervals (Table S1 in Additional file 1). The median of the average coverage for the samples was 187×. In all samples, 85% of the targeted bases were covered at 20× or more (Figure 2C). To perform sequence-based association studies, the consistency and reproducibility of coverage across targeted bases from sample to sample is of high importance. Coverage is directly correlated with accuracy in base calling and the same bases need to be analyzed across numerous samples. In general, targeted sequencing using LR-PCR provides good reproducibility, ensuring that any particular base will be covered equally well in different samples, provided there is a sufficient average coverage depth [25]. However, regions of high GC content are difficult to amplify and sequence [25] and in our current study are insufficiently covered in a number of samples (Figure 2A, B). Restricting the analysis to bases called in greater than 90% of the samples, we have 99.9% sensitivity to call homozygous bases (assuming a 3× coverage requirement) and 99.7% sensitivity to call heterozygous bases (assuming a 6× coverage requirement).

### Identification, filtering, and characterization of single nucleotide variants

We identified 1,448 single nucleotide variants (SNVs) that are polymorphic in the 289 sequenced samples using the MAQ SNP calling algorithm [26]. We implemented a number of quality filters to establish a reliable set of SNVs. We initially examined only the 1,433 SNVs that were biallelic, of which 1,403 (97.9%) were in Hardy-Weinberg equilibrium (HWE) at a *P*-value < 0.001 in the controls. The majority (19 of 27) of SNVs failing HWE had a lower than expected heterozygosity. Heterozygous genotypes in sequence data can be under-called for coverage or quality reasons. We observed a few cases where a 'hidden' variant (SNV or indel) was located in the vicinity of the SNV that failed the HWE test, leading to an erroneous call for alignment reasons. We imposed additional quality criteria where we assigned an 'N' genotype for a SNV covered by less than three reads or with poor consensus genotype quality (MAQ phred score < 10). Finally, we removed 16 SNVs for which less than 90% of the samples had valid genotype calls. These successive filters leave us with 1,393 SNVs confidently called in the sequenced cohort (Additional file 2). In addition to these 1,393 biallelic variants we also observed 5 tri-allelic variants (Table S2 in Additional file 1), of which 4 are private variants and observed only once (MAF = 0.002) and one is observed three times. This small number of tri-allelic variants (0.34% of the 1,448 SNVs) is consistent with the proportion of tri-allelic SNPs in the Seattle SNP database, which contains 67 tri-allelic SNPs (0.224%) [27]. For the biallelic SNVs identified, 433 of 1,393 (31%) are present in the dbSNP databases (v.129). Of the 960 (69%) novel SNVs, 512 (37%) were singletons (the minor allele was found only once) and 762 (55%) had a MAF < 1%. Since we sequenced 578 chromosomes, rare variants with a frequency of approximately 1% will be present in 6 chromosomes, and can thus be reliably identified. Our results demonstrate the power of deep population re-sequencing to discover rare variants (Additional file 3).

Coding variants are likely to have large effect sizes and their functional consequences can be predicted. We found 14 coding variants, of which 5 are common (MAF > 0.05) and 9 are rare (MAF < 0.003; observed only once or twice) (Table 1). Most of the common variants were previously known whereas the rare variants are novel. Of the 14 coding variants, 4 and 5 are non-synonymous coding variants in *FAAH *and *MGLL*, respectively, and 3 of them, all rare, are predicted to be damaging by SIFT [28]. Interestingly, rs324420, a coding allele, is predicted as tolerated despite evidence of its negative effect on FAAH enzymatic activity [19], thus showing the limitation of the predictive algorithm and underscoring the value of experimental validation by functional assays.

**Table 1:** Coding sequence variants in the two genes and SIFT analysis

Coordinate	Alleles	Gene	Codon change^a^	Amino acid change	dbSNP	Coding type	SIFT prediction	SIFT score	MAF	Number observed
Chr1_46643348	C/A	*FAAH*	CCA-aCA	P129T	rs324420	Non-synonymous	Tolerated	0.46	0.216	123
Chr1_46643944	G/A	*FAAH*	GGG-aGG	G226R	Novel	Non-synonymous	Tolerated	0.15	0.002	1
Chr1_46643960	C/T	*FAAH*	CCC-CtC	P231L	Novel	Non-synonymous	Tolerated	0.63	0.002	1
Chr1_46643996	G/A	*FAAH*	CGC-CaC	R243H	Novel	Non-synonymous	Damaging	0	0.003	2
Chr1_46644333	G/A	*FAAH*	GAG-GAa	E274E	Novel	Synonymous	Tolerated	0.96	0.052	30
Chr1_46644573	T/C	*FAAH*	TGT-TGc	C299C	rs324419	Synonymous	Tolerated	1	0.176	101
Chr1_46646834	G/A	*FAAH*	GCG-GCa	A356A	rs45476901	Synonymous	Tolerated	1	0.002	1
										
Chr3_128893754	C/T	*MGLL*	GCA-aCA	A307T	Novel	Non-synonymous	Tolerated	0.55	0.003	2
Chr3_128893854	A/C	*MGLL*	ATT-ATg	I273M	Novel	Non-synonymous	Tolerated	0.13	0.002	1
Chr3_128896571	T/C	*MGLL*	CTA-CTg	L251L	rs4881	Synonymous	Tolerated	1	0.073	42
Chr3_128922669	C/T	*MGLL*	GCA-aCA	A143T	Novel	Non-synonymous	Tolerated	0.36	0.002	1
Chr3_128983328	C/T	*MGLL*	GAC-aAC	D86N	Novel	Non-synonymous	Damaging	0	0.002	1
Chr3_129023325	C/T	*MGLL*	CGG-CGa	R19R	rs11538698	Synonymous	Tolerated	0.86	0.052	30
Chr3_129023335	G/A	*MGLL*	TCC-TtC	S16F	Novel	Non-synonymous	Damaging	0.01	0.002	1

^a^Changing nucleotide indicated as lower case.

### Quality assessment of the sequence-based genotypes

The use of next-generation sequencing for association studies is still an emerging field, and thus base-calling errors need to be better characterized to avoid confounding the association testing analysis. In particular, one needs to distinguish systematic errors due to the technology and random sampling errors due to low coverage. Here we use two separate assessment strategies to estimate the accuracy of our sequencing and define error types.

#### Comparison to an alternative genotyping method

To evaluate the accuracy of the filtered genotype calls using the sequence data, we independently genotyped 19 SNVs, present in dbSNP, in the two sequenced genes using the MassARRAY genotyping platform. We compared the sequence-derived genotypes for each sample to the corresponding MassARRAY genotypes and found that 1.8% (97 of 5,487 comparisons) of the genotypes were in disagreement between the two methods (Table 2). Sixty-four out of 97 (66%) of the discordant genotypes were located at three loci. Further inspection of these loci show that they are systematic errors due to the presence of a hidden un-annotated variant in the vicinity. The HWE statistic was higher for the MassARRAY genotypes at two loci, indicating that the MassARRAY genotyping was more often incorrect, likely due to the fact that the hidden variants were not considered during the primer design. Thirty out of 97 (31%) of the discordant genotypes were located in 10 of the remaining 13 loci. They were missed heterozygous in the sequence-based genotypes (N/N) and were likely a result of low sequence coverage and are thus random sampling errors. The last three discrepancies were due to missing genotypes. These results indicate that sequencing-based genotyping is more robust than MassARRAY genotyping to the presence of a hidden variant. A similar genotyping error type has been observed genome-wide with microarray genotyping, where 85 of 130 discrepant calls were due to 'hidden' SNPs [29]. This comparison shows us that 1.2% of all genotypes are discordant due to systematic errors in the genotyping platform whereas 0.6% are discordant due to low coverage or random sampling errors in the sequence data.

**Table 2:** Concordance of the sequence-derived genotype calls with genotypes from the MassARRAY genotyping for 19 SNPs

					Hardy-Weinberg statistic		
SNP rsID	Number matching genotype	Number of under-calls^a^	Number of over-calls^b^	Number of N/N^c^	Sequencing	MassARRAY	Hidden variant
rs594323	253	1	33	2	1.2	4.2	SNP at 22 bp
rs9759081	272	15	2	0	0	8.6	SNP at 19 bp
rs9852837	276	0	13	0	0.2	0.01	Indel at 38 bp
rs4141964	287	1	0	1	1.6	1.2	-
rs324419	287	2	0	0	0.7	0.9	-
rs17203666	289	0	0	0	0.6	0.6	-
rs11715363	286	3	0	0	0.1	0	-
rs17203659	288	1	0	0	0.2	0	-
rs6778770	287	0	1	0	0.2	0.3	-
rs17282181	283	4	2	0	0	0.2	-
rs497897	287	1	1	0	1.2	0.2	-
rs567384	283	4	2	0	2.3	0.6	-
rs3773155	286	3	0	0	0	0	-
rs3773159	286	1	2	0	2.3	2.1	-
rs13076593	288	1	0	0	0.8	0.1	-
rs936839	288	1	0	0	1.1	1.2	-
rs13066225	289	0	0	0	0.1	0.1	-
rs324420	289	0	0	0	0	0	-
rs7652615	289	0	0	0	0.4	0.4	-

^a^Genotype called as reference homozygote by sequencing and heterozygote by MassARRAY or heterozygote by sequencing and alternative homozygote by MassARRAY. ^b^Genotype called as heterozygote by sequencing and reference homozygote by MassARRAY or alternative homozygote by sequencing and heterozygote by MassARRAY. ^c^Uncalled genotype or tri-allelic in one of the two.

#### Comparison between replicate samples

**Figure 3:**
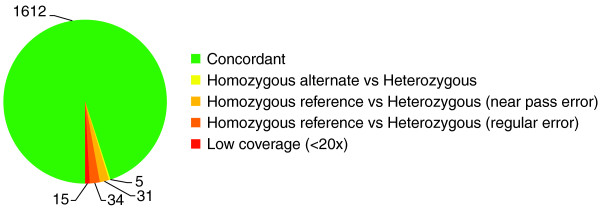
**Quality control of SNV identification**. Distribution of the matching status of 1,697 genotypes obtained from the 9 replicated samples.

The above comparison to an established genotyping method only assesses accuracy at well-behaved bases present in dbSNP. In order to assess all other bases as well as potential false positive variants, we compared sequence-based genotypes between independent duplicates of nine samples (independent library preparation and sequencing runs). We identified 448 SNVs present in one or more samples of 9 replicated samples; 429 of these passed the quality control filters established in the sequenced population, resulting in 1,697 pairs of genotypes to compare (most SNVs being present in more than one pair of duplicates). Of these, 1,612 (95%) pairs matched between the two replicates (Figure 3). Of note, the 5-kb regions upstream of *MGLL *and *FAAH *covered by single amplicons (Figure 2A, B) had 13 discrepant pairs; this increased error rate is likely due to the lower sequence coverage. Fifteen discrepant pairs had low coverage (< 20×) in one sample, which can create random sampling errors. Five discrepant pairs were homozygous alternative in one sample and heterozygous in the other. The remaining 65 pairs were heterozygous in one sample and homozygous reference in the other, of which 31 had some evidence of the alternative allele in the raw consensus call but failed Bayesian SNV caller (referred to as a near-pass error; see Materials and methods); 34 pairs did not show such evidence for the presence of an alternative allele. It is important to distinguish near-pass errors from regular errors since they can be rescued with optimized SNV calling or leveraging population information [30]. Our analysis reveals that in 289 samples, only 2.8% ((15 + 34)/1,697) of all variants were likely miscalled due to random sampling, whereas 2.1% ((31 + 5)/1,697) show an alternative allele under-calling, which was not sufficient to create Hardy-Weinberg disequilibrium. These data demonstrate that targeted sequencing using LR-PCR as the sample preparation method produces high sample-to-sample variant calling reproducibility.

### Detection of indels

The identification of insertions and deletions from short reads (36 bp) remains a challenge for two reasons: it is computationally prohibitive to align millions of short reads to a reference sequence allowing for gaps; and the alignments with indels are not reliable for short reads. The availability of paired-end reads alleviates the first problem since one end of the read can be anchored on the reference sequence and the second end can then be gap-aligned using a full Smith-Waterman alignment. According to previous reports, the SNV:indel ratio varies from 10:1 to 7:1 [29,31]; thus, we expect to find approximately 140 indels in the re-sequenced region. We used the MAQ *indelpe *module to perform paired-end mapping of the reads and to identify potential indel positions in each sample. This method identifies a large number of false positives and requires additional filtering to reliably call indels in the population. We identified 240 potential indel positions, 54 of which match an entry and allele call in dbSNP (v.129). Of the 240 indels, 106 are single base pair indels, 53 of them are located in homopolymer runs of 5 bp or longer and 24 in runs of 10 bp or longer, 21 indels are 2 bases long and, of these, 14 are located di-nucleotide repeats of length 2 or more; 143 indels pass HWE testing in the control samples, of which 49 match an allele in dbSNP. Interestingly, 5 indels failing HWE testing are *bona fide *variants present in dbSNP. The percentage of indels passing the HWE test (59.6%) is considerably lower than that of SNVs (97.9%), reflecting the difficulty to accurately call indels using short-read technology.

By sequencing 142 high BMI cases and 147 low BMI controls, we overall identified 1,393 high-confidence SNVs and 143 indels passing HWE testing for use in sequence-based association studies.

### Association of variants with BMI

As the sequenced samples were selected from the two tails of the BMI distribution, we performed association tests for each SNV with BMI as a binary trait to determine if any of the identified sequence variants in the *FAAH *and *MGLL *genes are associated with high BMI. We performed sequence-based association analysis using two different approaches: a chi-square analysis on all variants and a collapsing method for lower frequency variants.

#### Single marker tests

**Figure 4:**
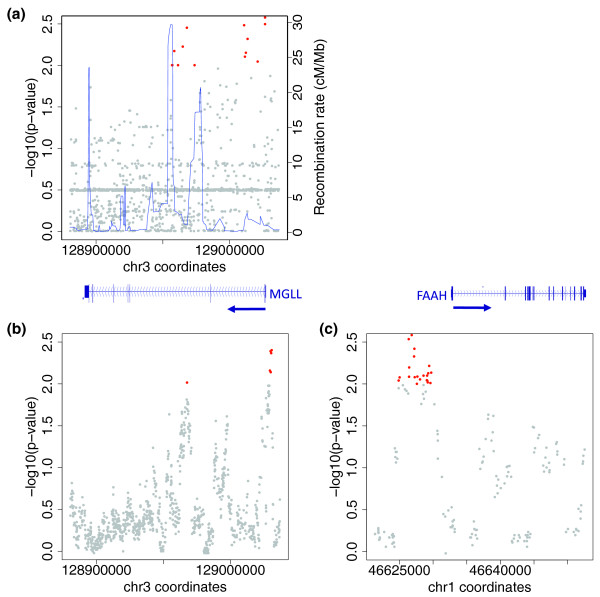
**Association with BMI**. **(a) **Significance of the association with BMI identified by single marker tests (-log10(chi-square *P*-value)) for all SNVs located in the *MGLL *interval (x-axis, NCBI36 coordinates). SNPs with a *P*-value < 0.01 are highlighted in red. The recombination rate [56] in the HapMap CEU population for this region is indicated by a blue line and measured on the right axis. **(b,c) **Significance of the association with BMI for all locus-variants identified by RareCover (see Materials and methods) in the *MGLL *(a) and *FAAH *(b) sequenced intervals. For both genes, locus-variants with a *P*-value < 0.01 are highlighted in red. The *MGLL *and *FAAH *gene structures are aligned based on their genomic positions.

We compared the allele frequencies of the variants in the cases and controls and assessed statistical significance using allelic chi-square test for each variant. Nineteen SNVs and one indel show an association with BMI (Table 3; chi-square *P*-value ≤ 0.01), of which 16 remain associated (*P *< 0.01) and 4 marginally associated (*P*-value approximately 0.01) after performing 5,000 permutation tests (Table 3). These associated variants are located in the non-coding part of the *MGLL *gene: three variants upstream, seven in intron 2 and ten in intron 3 (Figure 4A). The 20 associated variants are split between two linkage disequilibrium (LD) blocks demarcated by a recombination hotspot (Figure 4A) and could potentially affect regulatory elements located upstream or intronic to the gene. The variants in the left block have a lower frequency (MAF < 0.05) than the ones in the right block (MAF > 0.15). Interestingly, the risk effects of the minor alleles in the left and right blocks are opposite; most of the minor alleles in the right block are protective while most of those in the left block are associated with risk (Table 3). Of note, four of the associated variants were present on at least one of the genotyping arrays used in the original obesity GWASs (Table 3) but were not found associated with the trait. It is important to note that our study design, which is looking at extreme obesity (BMI ≥40 kg/m^2^) in an overweight population (mean BMI = 35 kg/m^2^), is different from most published GWASs, which missed the association at the *MGLL *loci.

**Table 3:** List of variants associated with high BMI by single marker tests

					MAF			Chi-square		Permutation
LD block	SNV-ID	Chr 3 coordinate	Gene location	Minor/major alleles	Cohort	Cases	Controls	*P*-value	OR	*P*-value
Left	rs16830415	128956957	Intron3	C/T	0.028	0.045	0.010	9.95E-03	4.59	8.00E-03
	Chr3_128957192	128957192	Intron3	G/T	0.028	0.045	0.010	9.95E-03	4.59	8.00E-03
	Chr3_128958587	128958587	Intron3	C/T	0.043	0.066	0.021	6.70E-03	3.39	5.00E-03
	Chr3_128958866	128958866	Intron3	-/T	0.08	0.049	0.1103	7.07E-03	0.41	7.40E-03
	rs9832418	128961356	Intron3	C/T	0.028	0.045	0.010	9.95E-03	4.59	8.00E-03
	rs547801^a^	128964929	Intron3	T/C	0.029	0.049	0.010	5.93E-03	4.96	5.00E-03
	rs520154^a^	128965687	Intron3	A/G	0.028	0.049	0.007	2.04E-03	7.46	1.20E-03
	rs60963555	128967982	Intron3	T/C	0.026	0.045	0.007	3.52E-03	6.91	1.60E-03
	rs684358^b^	128969940	Intron3	G/T	0.028	0.049	0.007	2.04E-03	7.46	1.20E-03
	rs9852837	128973744	Intron3	A/G	0.028	0.045	0.010	9.95E-03	4.59	1.16E-02
										
Right	rs9289319	129009856	Intron2	G/A	0.192	0.138	0.243	1.42E-03	0.50	1.80E-03
	rs9289320	129010946	Intron2	G/C	0.192	0.143	0.240	3.27E-03	0.53	6.00E-03
	rs9289321	129011459	Intron2	A/G	0.165	0.123	0.206	7.84E-03	0.54	9.80E-03
	rs9877819^c^	129012220	Intron2	A/G	0.164	0.122	0.206	7.03E-03	0.54	7.40E-03
	rs28753886	129013477	Intron2	A/G	0.163	0.119	0.206	4.79E-03	0.52	5.60E-03
	rs35948688	129014938	Intron2	C/T	0.159	0.112	0.206	2.10E-03	0.49	2.40E-03
	rs874546^c^	129021102	Intron2	G/A	0.183	0.140	0.226	8.99E-03	0.56	1.00E-02
	rs2011138	129026619	Upstream	A/C	0.352	0.412	0.295	3.18E-03	1.68	4.40E-03
	Chr3_129026621	129026621	Upstream	A/G	0.049	0.021	0.075	2.65E-03	0.27	4.00E-03
	Chr3_129029015	129029015	Upstream	A/G	0.336	0.398	0.276	1.98E-03	1.74	3.60E-03

^a^Present on the Affymetrix 500 k genotyping array. ^b^Also part of a locus-variant associated with high BMI using the collapsed marker test RareCover (Table S5 in Additional file 1). ^c^Present on the Illumina HumanHap300 genotyping array. LD, linkage disequilibrium.

Several other SNPs located in *FTO *[6,32-35], *MC4R *[7,36,37], *CNR1 *[11,38,39], *CTNNBL1 *[8], *INSIG2 *[40] or *PFKP *[35] have been associated with high BMI or obesity by GWASs. In order to relate these previous results to the population in our study, we genotyped the associated SNPs in the 289 individuals we sequenced. Looking at BMI as a binary trait, we found that all the SNPs located in *FTO *were associated with high BMI (*P*-value < 0.05; Table S3 in Additional file 1). None of SNPs located in the other genes showed association with high BMI. These results demonstrate that despite differences in the sample selection criteria, our cohort is appropriate to replicate the association of variants in the *FTO *gene interval, one of the strongest associations in recent obesity GWASs. In a recent and remarkable meta-analysis of the majority of published obesity GWASs, the authors show that the replication of the *INSIG2 *locus association was compromised by study design [41]. Thus, the failure to replicate originally weaker associations in our study and the failure to identify *MGLL *in previous GWASs can be due to insufficient power, population differences, variable study designs or selection criterion.

#### Collapsed marker tests with RareCover

Statistical association with single variants of low allele frequency is challenging to assess as very few samples contribute to the association test. Previous studies have used collapsing methods to study the influence of rare variants on high-density lipoprotein plasma levels [42], colorectal cancer risk [43] or type 1 diabetes [44]. More recent collapsing methods use a weighted or multivariate model. Here, we implement a model-free method (RareCover [24]; see Materials and methods) to identify an optimal set of variants of low allele frequency (MAF ≤ 0.1) within a moving 5-kb window, which maximizes the association with high BMI. We refer to variants in the 5-kb window as locus-variants. This strategy increases the power of detecting an association using variants of low allele frequency with moderate relative risk and cohort sizes.

Using RareCover on the low frequency SNVs (MAF < 0.1, indels excluded), we identified 31 locus-variants in the *FAAH *and *MGLL *interval that are significantly associated (permutation *P*-value < 0.01; Table S4 in Additional file 1) with extreme obesity (Figure 4B, C). Most of these locus-variants are overlapping and share several SNVs; however, three distinct intervals show significant association with high BMI. The first interval is located in the *FAAH *promoter region. The most significant locus-variant of this interval harbors 15 variants selected by RareCover for maximizing the association (permutation *P*-value = 2.2 × 10^-3^; Table S4 in Additional file 1). Twenty-three cases and no controls carry a minor allele at the union of the 15 variants (Table S5 in Additional file 1). The second interval is located in the *MGLL *promoter region. RareCover identified 10 variants (permutation *P*-value = 1.4 × 10^-3^) in the most significant locus-variant of this interval; 38 cases and 9 controls carry a minor allele at the union of the 10 variants (Tables S4 and S5 in Additional file 1). Thus, for both genes, the most significantly associated locus-variants are located upstream of the transcription start sites with potential consequences on the regulation of gene expression. Because these upstream regions have lower coverage due to their amplification by a single amplicon (Figure 2A, B), we verified that all SNV alleles found associated with BMI, either by the single marker or the RareCover collapsing method, has sufficient coverage (Table S8 in Additional file 1) to generate reliable genotypes. Finally, the third interval is located in *MGLL *intron 3 and overlaps with the left block SNVs associated with high BMI by single marker analysis (Figure 4A, B). It has only one significant locus-variant (*P*-value = 0.0096) consisting of 9 variants; 25 cases and 2 controls carry a minor allele of the union of the 9 variants (Table S5 in Additional file 1). One of the nine variants in the *MGLL *intron 3 locus-variant (rs684358) was also identified as associated with high BMI in the single marker analysis. Interestingly, the eight other variants associated with BMI using single marker analysis are not included in the reported significant locus-variants. This is due to the fact that these variants are in LD and thus the associated alleles are carried by the same individual: their addition in the RareCover locus-variant would not change the *P*-value and thus they were not included. Although these eight variants are included in some other locus-variants, the *P*-value does not reach significance since its calculation differs from the single marker test by the inclusion of other variants and the finite number of permutations. The second most associated variant by the single marker test (rs520154) is included in a mildly significant locus-variant (*P*-value approximately 0.03); the seven other variants had a higher single-marker *P*-value. Of note, the right block identified by the single marker analysis harbors only more common variants (MAF > 0.15), which were not included in the RareCover analysis. Thus, in the same interval of *MGLL *intron 3, both the single marker and RareCover tests independently identified variants with different MAFs (approximately 0.03 versus approximately 0.002) that are associated with high BMI.

### Functional annotation of the associated variants

**Figure 5:**
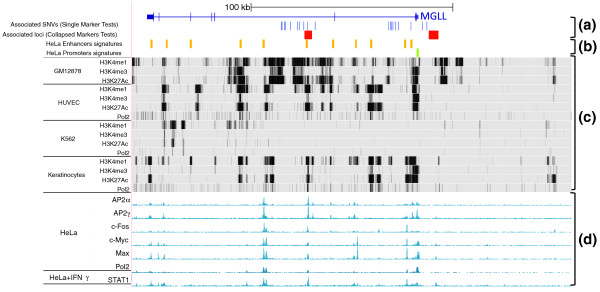
**Functional annotation of the associated variants in the *MGLL *interval**. Track A: variants identified by the single marker tests (blue bars) or the merge of the locus-variants identified by the collapsed marker test RareCover (red boxes) are indicated. Track B: predicted intervals for promoters (green) or transcriptional enhancers (orange) in HeLa cells [45]. Tracks C and D: the distribution of chromatin binding proteins from the ENCODE data obtained from the UCSC genome browser (15 November 2009). Track C: Broad/MGH ENCODE group chromatin signatures corresponding to enhancers (H3K4me1, H3K27ac) and promoters (H3K4me1+3, Pol2) in various cell types (GM12878, HUVEC, K562, Keratinocytes) [46]. Track D: Yale/UCD/Harvard ENCODE group identified AP2α and γ, c-Myc, Max, c-Fos and Pol2 binding sites in HeLa cells, and STAT1 binding sites in HeLa treated with IFNγ [46].

DNA variants located outside of coding regions can lie in transcriptional regulatory elements and have an effect on gene expression. In order to determine the potential regulatory function of the variants or locus-variants associated with high BMI, we inspected publicly available chromatin marks around the *MGLL *and *FAAH *genes. In particular, the combined location on the DNA sequence of several histone modifications as well as transcriptional co-activators and RNA polymerase has been used in HeLa cells to determine genome-wide signatures for transcriptional enhancers and promoters [45]. Interestingly, the *MGLL *interval has 11 predicted enhancers in HeLa cells; however, there are no predicted enhancers in the *FAAH *interval (Figure 5, track B). The locus-variant identified by RareCover in *MGLL *intron 3 and also identified via single marker test (Figure 5, track A) overlaps an enhancer prediction. Chromatin marks corresponding to this particular enhancer are also identified in several cell types studied by the ENCODE consortium [46] (Figure 5, track C). In addition, a number of transcription factors bind this particular element in HeLa cells as shown by the ENCODE consortium [46] (Figure 5, track D) adding further evidence that it is likely to be an enhancer. Since enhancers can be active in multiple cell types, it is very likely that the variants associated with high BMI in *MGLL *intron 3 affect the activity of a transcriptional enhancer by modifying a transcription factor binding site, thus changing *MGLL *gene expression in the central nervous system or other, peripheral tissues. Similarly, one of the single associated SNVs in *MGLL *intron 2 also lies in an enhancer prediction. This particular SNV could well be associated with high BMI because of its causal regulatory role in *MGLL *expression while the other SNVs in the right block could be associated because of their LD with it. Interestingly, none of the associated variants are present in evolutionarily conserved sequences, which frequently are a signature for regulatory elements. These analyses suggest that two of the intervals (*MGLL *intron 2 and intron 3) associated with high BMI contain regulatory variants in enhancer elements.

### Consequences of associated variants on EC levels

Reduced levels of FAAH and MGLL catabolic enzymes can lead to an accumulation of their substrates AEA and 2-AG, respectively. In an attempt to link the presence of the associated alleles in high BMI patients to the level of circulating EC, we measured the plasma concentrations of AEA and 2-AG in a subset of the samples. We selected 96 obese patients with BMI > 45 kg/m^2 ^and 48 normal patients with BMI < 26 kg/m^2 ^and measured the concentration of AEA and 2-AG in the plasma using reverse phase liquid chromatography coupled to triple-quadrupole mass spectrometry (TQMS). We calibrated our measurements by comparison to deuterated standards.

None of the single variants located in *MGLL *and associated with high BMI showed a significant association with either AEA or 2-AG levels. Examining the most significantly associated locus-variants from each of the three intervals identified by RareCover, we compared AEA and 2-AG average levels between carriers in the obese samples versus non-carrier control samples (Table 4). Case individuals carrying the locus-variant minor alleles in *FAAH *had significantly higher levels of AEA (+24%) than control non-carrier individuals (*t*-test *P*-value = 0.05), with a consistent trend across all classes (carrier/cases, non-carrier/cases, non-carriers/controls) (Figure S2 in Additional file 4). This trend is consistent with the higher observed levels of AEA in obesity [16], which could result from reduced expression of *FAAH *in some obese individuals because of rare variants in the promoter region (Table 4).

**Table 4:** Average endocannabinoid levels of FAAH and MGLL rare variant carrier groups at the most significant locus-variants in the three intervals

	AEA				2-AG			
		
	N	Average (pmol/ml)	SD	***P*-value**^**a**^	N	Average (pmol/ml)	SD	***P*-value**^**a**^
*FAAH *promoter locus-variant				0.05				0.10
Carrier								
Cases	14	17.11	5.79		14	10.55	10.67	
Controls	0	NA	NA		0	NA	NA	
Non-carrier								
Cases	80	15.13	5.34		67	5.63	3.04	
Controls	48	13.76	5.51		35	6.98	4.15	
								
*MGLL *intron 3 locus-variant				0.49				0.80
Carrier								
Cases	5	15.6	6.35		4	6.43	2.08	
Controls	0	NA	NA		0	NA	NA	
Non-carrier								
Cases	89	15.24	5.44		77	6.49	5.57	
Controls	48	13.76	5.52		35	6.99	4.16	
								
*MGLL *promoter locus-variant				0.36				0.37
Carrier								
Cases	26	15.09	5.6		24	6.31	3.06	
Controls	3	13.16	5.38		3	4.41	4.46	
Non-carrier								
Cases	68	15.56	5.43		57	6.56	6.2	
Controls	45	13.8	5.58		32	7.22	4.11	

^a^Two-tailed *t*-test *P*-value between carriers/cases and non-carriers/controls. NA, not available.

## Conclusions

In this study, we generated high quality sequencing data to analyze the association of DNA variants in two candidate genes, *FAAH *and *MGLL*, with extreme obesity. Deep population sequencing allows one to test for the association of alleles spanning the entire frequency spectrum. By using two different approaches, single marker tests and collapsed marker tests, we were able to identify one interval in the *FAAH *promoter and three intervals in the *MGLL *gene, one each in the promoter, intron 2, and intron 3, all associated with high BMI. Most of the associated variants are rare (MAF < 0.01) or have low frequencies (MAF ≈ 0.03) and are only accessible via population sequencing. The single-base-pair resolution obtained in the sequencing-based association study allowed us to precisely map the associated variants to a predicted transcriptional enhancer in HeLa cells or to the promoter regions. Thus, the associated variants are likely regulating the expression of *MGLL *and *FAAH*. By potentially affecting the overall transcription rate of the two genes, the variants can influence the EC degradation rate. A correlation between decreased expression of *FAAH *in adipose tissues and increased circulating AEA levels has previously been observed in obese patients [16]. The expression of *FAAH *and *MGLL *in numerous tissues will make it challenging to determine the exact role that the regulatory variants identified in our study play in obesity.

Our study design examines extreme cases of obesity in an overweight population. We demonstrate the ability to replicate in our population the association of *FTO *variants with obesity, which is the main BMI-associated locus, thus further proving the robustness of the association and the appropriate selection of our samples to study obesity. However, some other loci, more weakly or inconsistently associated in the original GWASs, were not replicated in our samples, which is not too surprising given the sample size of our cohort is inadequate to replicate modest associations. Reciprocally, the published GWASs did not find any association in *FAAH *or *MGLL *despite the presence of probes for 4 out of 20 single marker-associated variants in the genotyping microarrays used. This lack of consistency has been studied through a meta-analysis of obesity GWASs [41] in which replication was compromised by the type of population sampled, the BMI thresholds used for cases and controls, the fraction of obese people, or the time of study reflecting change in the environment. This study highlights the crucial importance of population and study design in obesity association studies for both discovery and replication. It is important to note that, in our study, we strengthen the initial genetic association by correlating it to its functional consequences in both *FAAH *and *MGLL*. Using metabolite measurements in the plasma of the sequenced samples, we verified that the set of rare variants in the *FAAH *promoter associated with high BMI is also associated with an increased level of AEA. Additionally, we demonstrate independent associations of common and rare variants in *MGLL *intron 3 and show that these variants overlap a predicted transcriptional enhancer, which suggests their regulatory role.

The large number of loci identified by GWASs only explains a small fraction of the estimated heritability underlying complex diseases. Since GWASs using arrays examine only common variants for association, it is possible that rare variants comprise an important component of the hidden heritability. Compared with previous GWASs, sequencing-based studies can examine rare variants for association with complex traits. It is believed that a significant fraction of the heritability missed by GWASs lies in rare variants. Similar to our approach, other studies have collapsed rare variants from several samples to assess a significance difference in frequency between two groups [42,44]. Although the effect size of rare variants cannot be accurately estimated in a relatively small cohort, it has been shown that they contribute to an incremental fraction of heritability in hypertrigyceridemia [47]. Our study is the first to use this approach on contiguous genomic intervals and not only in coding regions. This allows the identification of potential regulatory variants. Most common variants found in GWASs of common diseases lie in non-coding regions, often very distant from genes. These variants, or variants in LD with them, are thought to affect regulatory elements, as some studies have demonstrated [48,49]. The effect of the rare variants in common diseases might be similar and more frequently affecting regulatory elements: this hypothesis fits particularly late-onset or chronic disease etiology in which the symptoms can be the result of long-term mild imbalance in the regulation of molecular functions. As sequencing technology and bioinformatics tools improve, we will be able to reliably call copy-number variation in large cohorts and consider gene interactions to explore even further the missing heritability.

In order to improve the sensitivity of genetic association studies, the biology underlying the complex phenotype also needs to be considered. For this purpose, the epigenetic landscape, such as chromatin marks, is particularly important to identify the functional variant from a group of associated variants all in LD and go beyond the pure genetic assessment of disease susceptibility. Knowledge of the biochemical activity of the gene products is also helpful. For example, the extensive annotation of metabolic pathways constitutes a powerful paradigm with a direct measurable output. We sequenced two genes coding for metabolic enzymes important for the regulation of the EC system, and looked upstream of their consequences on BMI, at the level of their substrates, to confirm their influence. Thus, an integrated approach using deep sequencing to find rare variants, epigenetic annotation of the DNA sequence and functionally relevant endo-phenotypes increases the odds of finding elements of missing heritability and helps to more fully comprehend the underpinnings of complex disease etiology. With increasing availability of large scale functional datasets, integrated approaches such as ours will likely become more common in future genetic association studies.

## Materials and methods

### Selection of samples for sequencing

The Institutional Review Board of Sanofi Aventis approved the collection of samples from the CRESCENDO cohort [50] and the unrestricted release of the results of this study. The enrollment of participants and blood collection were carried out in accordance with the Helsinki Declaration. In particular, patients gave informed consent to the study. An initial list of 3,101 individuals in the CRESCENDO cohort were evaluated and filtered to select low and high BMI/obesity samples, representing the two extremes of this phenotype, for both genders. Individuals of European ancestry are highly represented (96%) in the CRESCENDO cohort. To reduce false-positive findings due to differing genetic backgrounds, male and female selection was restricted to individuals of European ancestry, ranging in age from 55 to 77 years. Samples with inconsistent or aberrant measurements were removed from the set, including 52 samples from patients with inconsistent waist measurements (standard deviation > 3), 2 samples with missing biographical data, and from one subject with a nonsensical BMI (BMI = 195). For the low BMI/obesity sample subset, male and female subjects with a BMI of ≤ 30 kg/m^2 ^were selected. For the high BMI/obesity sample subset, male and female subjects with a BMI of ≥40 kg/m^2^, but < 60 kg/m^2^, were selected to remove aberrant outliers. The last criterion used for selection was the waist measurement. First, the BMI measurements were plotted against the average waist measurements, and two outliers on the BMI/waist measurement graph were removed. Based on availability and quality of DNA, a subset of each category was selected for deep population sequencing: 73 men and 70 women with a BMI > 40 kg/m^2 ^and 74 men and 72 women with a BMI < 30 kg/m^2 ^(Figure 1). DNA was isolated from whole blood collected from each of the 289 selected individuals.

Differences in ancestry between cases and controls can lead to spurious associations in case-control association studies. Although all individuals in the CRESCENDO cohort that were selected for sequencing have self-reported European ancestry, we utilized a set of ancestry informative markers to ensure that the sequenced samples have primarily European ancestry; 31 ancestry informative markers chosen from the Human Diversity Panel [51] were genotyped in the 289 samples using the Sequenom MassARRAY genotyping platform. We performed principal components analysis of the genotype data [52] using Matlab (MathWorks, Natick, MA, USA) and only the first principal component was significant, indicating lack of population structure.

### Long-range PCR

Forty LR-PCR experiments were performed to amplify 31,716 bp encompassing the *FAAH *gene (NCBI36 chr1:46621328-46653043) and 156,556 bp encompassing the *MGLL *gene (NCBI36 chr3:128880456-129037011). We performed the 40 LR-PCR experiments using 5 ng of genomic DNA, 0.5 μM forward LR-PCR primers, 0.5 μM reverse LR-PCR primers (Table S6 in Additional file 1) in a total reaction volume of 12 μl, as described [2]. Following LR-PCR, the 40 amplicons (3,129 bp to 12,203 bp) generated using a single DNA sample template were quantified using Quant-IT technology (Invitrogen, Carlsbad, CA, USA) and combined in equimolar amounts using a liquid handling robot (Biomek NX; Beckman Coulter, Brea CA, USA).

### Illumina GAI library preparation

The following steps were performed in 96-well microtiter plates unless otherwise specified. The pooled amplicons (1 μg) were fragmented to an average size of 200 bp (between 170 and 250 bp) using 0.005 U of DNase I for 15 minutes at 37°C followed by an inactivation step of 10 minutes at 99°C. The fragmented DNA was purified in a Qiaquick 96 PCR purification plate (QIAGEN, Valencia, CA, USA) following the manufacturer's instructions. The DNA ends were repaired in a 100 μl reaction (1× NEB ligase buffer, 1 mg/ml bovine serum albumin, 200 μM dNTP) using 15 U T4 DNA polymerase (NEB Ipswich, MA, USA), 50 U T4 polynucleotide kinase (NEB), and 5 U Klenow DNA polymerase. The reaction was incubated for 30 minutes at 20°C and the DNA purified on a Qiaquick 96 PCR purification plate. The 3' end was extended with a single overhanging A using 15 U Klenow exo-, 200 μM dATP in 50 μl NEB2 reaction buffer and incubated for 30 minutes at 37°C. The DNA was purified on a Qiaquick 96 PCR purification plate. The eluted DNA was mixed with 10 μM of indexed adapters (see sample indexing below and Table S6 in Additional file 1) and ligated for 15 minutes at room temperature with 2,000 U DNA ligase in 50 μl ligase buffer (NEB) followed by purification on a Qiaquick 96 PCR purification plate. The DNA was separated from the free adapters by 2% agarose gel electrophoresis, the smear ranging from 120 to 210 bp was extracted, melted in 3 μl QG buffer per milligram of gel for 20 minutes at 50° in a 800 μl deep well micro-titerplate and purified using a Qiaquick 96 PCR purification plate. The adapter ligated fragments were then enriched by PCR using the library enrichment primer pairs (Table S6 in Additional file 1) in a 50 μl PCR containing 4 μl template DNA, Phusion HF buffer (NEB), 200 μM dNTP, 0.4 μM of both Solexa primers, 3% DMSO, 0.5 μl Phusion DNA polymerase. The PCR involved denaturing for 5 minutes at 98°C followed by 20 cycles of 10 s at 98°C; 20 s at 65°C, 15 s at 72°C, and a final elongation of 4 minutes at 72°C. The DNA library was then purified on a Qiaquick 96 PCR purification plate.

### Sample indexing and sequencing

In order to sequence several samples per lane on the Illumina GA flow cell, we implemented an indexing strategy similar to that described in Craig *et al*. [53]. We generated 12 pairs of modified DNA adaptors with 4 nucleotide DNA barcodes at the 3' ends; the first and last nucleotides of the DNA barcode were constant while the two middle nucleotides vary (CNNT; Table S6 in Additional file 1). Both strands of the indexed adapter (Integrated DNA Technologies Coralville IA, USA) were mixed at 100 μM in TE pH 8.0, denatured for 5 minutes at 95°C, placed in a heat block at 70°C, left at room temperature until reaching 25°C, and then transferred to 4°C and left overnight, enabling the two strands to anneal. The annealed strands were then stored at -20°C.

The libraries were quantified by Quant-IT technology (Invitrogen) in quadruplicate, diluted to 10 nM. From one to seven indexed libraries were combined together into one pool, denatured with NaOH, and then 2.3 pM of each pool was loaded into one lane of an Ilumina GA flow cell and sequenced using Illumina Single-Read Cluster Generation Kit v1 and SBS Kit v1 for 40 cycles, thus providing 36-nucleotide reads after removal of the 4 nucleotides used for DNA barcoding. All the sequencing data are publically available from NCBI short read archive study #SRP003433.

### Image analysis pipeline, read alignment and variant calling

We used the Illumina Pipeline version 1.0 to analyze the raw images, masking the first four bases of the index (USE_BASE option IIIIY*) for accurate base call calibration. After base calling, quality calibration and read filtering, Python scripts were used to parse out the indexes and create the sequence files for each sample. For paired-end reads, we used the index of the high quality first read to assign the read to a particular sample, regardless of the index from the lower quality second read (matching in > 95% of the cases).

We used MAQ (version 0.6.8) [26] to align the reads to the reference sequence allowing for three mismatches in the first 24 bp (maq map -n 3). The 289 sample libraries were sequenced across multiple runs of the Illumina GA, 250 samples were sequenced with only paired-end runs, 38 samples were sequenced with at least one paired-end run, and one sample was sequenced in single reads only. For samples sequenced multiple times (with the exception of technical replicate samples of the MultiQC set; see below), the mapped reads were merged to create a single set of mappings for each sample (maq mapmerge). The MAQ variant calling method was used to call variants using default parameters (maq.pl SNPfilter was used to filter out false positives) for each sample. MAQ assigns a most likely genotype for each site and detects potential SNVs in each individual. The set of variants across all 289 samples was combined to create the list of 1,451 raw variants. For each SNV site, we used the MAQ cnsview files to determine the genotype for each sample and the coverage and consensus quality at every position. Genotypes with a quality score below 10 or covered by less than three reads were assigned the NN genotype.

### Detection of insertions/deletions

MAQ outputs all read alignments with indels. We used the filtered *indelpe *files as the initial set of potential indels. We used the following steps to determine a reliable set of indels from the *indelpe *files. Step 1, we merged the set of indels reported by MAQ for all 287 samples (2 samples did not have enough paired-end reads). Step 2, we clustered together the indels in multiple samples based on the position of the indels in the reference sequence. This is required since indels located in homopolymer stretches of sequence can have multiple starting locations. Step 3, for each individual, we assigned the genotype for an indel as heterozygote if the proportion of the non-reference reads was between 0.2 and 0.8. Step 4, the genotype was assigned as reference homozygote and alternative homozygote if the proportion was less than 0.2 and more than 0.8, respectively. Step 5, for each indel detected in the population, we required at least one sample to have three reads with the indel variant and at least eight reads covering the variant site. We imposed stringent cutoffs for rare indels (MAF < 0.01). For such indels, we required coverage of at least 10 with 5 reads containing the indel variant.

### Genotyping HapMap SNPs

The SNPs were genotyped using the Sequenom MassArray genotyping platform. We selected 19 HapMap CEU tag SNPs from the two gene regions (3 in *FAAH *and 16 in MGGL; Table S7 in Additional file 1). PCR assays and extension primers for these SNPs were designed using the MassARRAY Assay Design software, version 3.1 (Sequenom San Diego CA, USA). SNPs were genotyped using the iPLEX Gold assay, based on multiplex PCR followed by a single base primer extension reaction. The mass of the primer extension products, correlating to genotype, were determined using matrix assisted laser desorption/ionization time-of-flight (MALDI-TOF) mass spectrometry. Final genotypes were called using the MassArray Type, version 4.0.

### Genotyping obesity SNPs

The genotypes were determined after PCR and 5' nuclease assay (allelic discrimination with ABI TaqMan specific probes) reaction and read on an ABI 9700 (LIFE Technologies, Carlsbad, CA USA) The PCR primers and probes were chosen according to the supplier (LIFE Technologies, Carlsbad, CA USA). All reagents and software used are licensed to Applied Biosystems. The genotypes were analyzed on an ABI7900 automated sequencer and determined using the SDS2.0(r) allelic discrimination software. No deviations from Hardy-Weinberg proportions were detected; the genotyping failure rate was 1%.

### Sample sets for quality control

The nine samples were processed as blind replicates, meaning each replicate was treated as an entirely separate sample with independent PCR amplification, library preparation and sequencing as well as analysis. The resulting genotype pairs were compared, assigning the following matching status: 1, concordant between replicates; 2, heterozygous versus homozygous alternative; 3, heterozygous versus homozygous reference with evidence of alternative allele in the raw data (second most likely genotype - near-pass error); 4, heterozygous versus homozygous reference without evidence of alternative allele; 5, low covered position (< 20×) in one of the replicates resulting in erroneous genotypes.

### Single marker association

The test for association was performed using PLINK1.06 [54]. The SNPs were filtered for HWE in the controls and genotyping rate > 0.9. We performed a binary case/control association using a chi-squared test (--assoc), considering high BMI samples as cases and low BMI samples as controls. The max(T) permutation *P*-value (--mperm 5000) was obtained after performing 5,000 sample label permutations.

### Association with rare variants

We used the RareCover algorithm described in Bhatia *et al*. [24]. Briefly, we define S as the set of rare variants (MAF ≤ 0.1) present at a locus L_S_, which is a window of size 5 kb. RareCover examines overlapping windows, where each window is shifted one rare variant away from the previous rare variant. We define C as a subset of S composed of rare variants that contribute to *A_C_*, the union-variant, a virtual construct that combines the effects of multiple rare variants. The variants in C together form a locus-variant L_C_, and for an individual sample *A_C _*= 1 if at least one of the variants carries the minor allele, and otherwise *A_C _*= 0. By using a chi-square statistic test between high and low BMI samples at the locus L_C_, we find the optimal subset of rare variants C for which the association is maximal, thus defining the test-statistic for the locus L_S_. The level of significance was obtained by performing 10^4 ^randomizations of the data set, permuting cases and controls, and re-computing the test-statistic for the selected locus-variant with the permuted samples. A locus L_S _is considered significant if the permuted *P*-value is < 0.01. To correct for the total number of locus-variant windows tested per gene, another 10^6 ^permutations of cases and controls were performed and used to evaluate the significance of all locus-variants in the gene [24]. The three locus-variants reported in our study all had a *P*-value ≤ 0.05 when corrected for the number of windows tested.

### Evolutionarily conserved sequences

Conserved bases were defined as nucleotides with a conservation score ≥0.1758 (5th percentile in the interval) in the multispecies sequence comparison track at UCSC (28 way placental mammals PhyloP conservation score).

### Measurement of endocannabinoid levels in plasma

Whole blood samples were collected in evacuated glass tubes containing EDTA. Samples were centrifuged to separate plasma from blood cells and plasma was withdrawn and stored in 1-ml aliquots at -80°C prior to plasma lipid extraction. For each sample, 0.5 ml plasma was added to a glass vial containing 2.0 ml chloroform (CHCl_3_), 1.0 ml methanol (MeOH) and 0.5 ml (1% v/v) formic acid. To this mixture were added aliquots of 10 pmol D5-2-arachidonlyglycerol (2-AG) and 5 pmol D8-arachidonlyethanolamine (AEA). Vial contents were vortex mixed for 30 s and centrifuged at 10°C (1400 × g for 10 minutes). The organic layer was carefully removed avoiding the aqueous layer and dried under a stream of nitrogen (N_2_) gas. The lipid layer was then re-solubilized in 100 μl of 2:1 CHCl_3_:CH_3_OH.

Quantitative analysis of EC metabolites using the deuterated standards for AEA and 2-AG together with calculation of the other EC metabolites was based on a ratio to the deuterated standards and was performed on an Agilent 6410 liquid chromatography triple-quadrupole mass spectrometer using positive ion analysis mode. For each sample, 20 μl of re-solubilized plasma lipids were injected into the TQMS and EC metabolites were measured by multiple reaction monitoring using the following transitions: 348 > 62 (AEA), fragmentation energy = 8; and 379 > 287 (2-AG), fragmentation energy = 11. Chromatography was performed using the following solvents: A, 95:5:0.1 H_2_O:methanol:formic acid; and B, 60:35:5:0.1 isopropanol:methanol:H_2_O:formic acid. Lipids were injected into a 5 micron particle size C18 column (50 × 4.6 mm) from Phenomenex (Torrance, CA, USA) and eluted with a 10-minute solvent B gradient from 60% to 100%. Values for each EC metabolite were subsequently calculated using ratios to the deuterated internal standards to calculate absolute concentrations, expressed as picomoles (pmol) of EC metabolite per milliliter of plasma similar to current methods [55].

## Abbreviations

2-AG: 2-arachidonoylglycerol; AEA: anandamide; BMI: body mass index; bp: base pair; EC: endocannabinoid; FAAH: fatty-acid amide hydrolase; GWAS: genome wide association study; HWE: Hardy-Weinberg equilibrium; indel: insertion-deletion; LD: linkage disequilibrium; LR-PCR: long range PCR; MAF: minor allele frequency; MGLL: monoglyceride lipase; SNP: single nucleotide polymorphism; SNV: single nucleotide variant; TQMS: triple-quadrupole mass spectrometry.

## Authors' contributions

OH and VB performed the sequencing and statistical analysis. GB and VB performed the rare variant analysis. MS and JS performed the mass spectrometry experiments. OH, XW and MN designed and performed next-generation sequencing experiments; SM designed and performed the MassARRAY genotyping. JFD, CD, ET provided the samples and performed genotyping experiments. KAF, EJT, JFD, SM and OH designed the study. KAF and OH wrote the manuscript.

## Supplementary Material

Additional file 1 **Supplementary tables**. Click here for file

Additional file 2 **Supplementary Figure S3**. Flowchart illustrating the filtering steps for the variant calling. Click here for file

Additional file 3 **Supplementary Figure S1**. Distribution of the minor allele frequencies in the sequenced population for SNVs present in dbSNP (light grey) or novel SNVs (dark grey) in the *FAAH *and *MGLL *sequenced intervals. Click here for file

Additional file 4 **Supplementary Figure S2**. Average AEA plasma levels (pmol/ml) in 48 non-carriers controls, 80 non-carrier cases and 14 case carriers of the most significant *FAAH *variant-locus allele associated with high BMI. Error bars represent the standard deviation from the mean. Click here for file
