# Supplementary material for: Population sequencing of two endocannabinoid metabolic genes identifies rare and common regulatory variants associated with extreme obesity and metabolite level
Source: Genome Biol. 2010 Nov 30;11(11):R118. doi: 10.1186/gb-2010-11-11-r118 (PMC3156957; doi:10.1186/gb-2010-11-11-r118)
Supplement: Additional file 1 — Supplementary tables. [file gb-2010-11-11-r118-S1.PDF]

Supplemental Table 1 : Sequencing Reads and Coverage data for 289 sequenced samples

Sample ID	Gender	BMI (kg/m2)	Avg. Coverage	Std Dev.	Base Pairs	Mapped Reads	Mapped in pairs	Fraction of Bases > 20x	Fraction of Bases > 50x	Fraction of Bases > 80x
J059269	FEMALE	25	179.41	178.89	188270	1019743	747872	0.975	0.863	0.729
J059349	FEMALE	29	221.37	227.72	188270	1263552	957394	0.984	0.917	0.802
J059386	FEMALE	49	221.11	227.93	188270	1237874	914494	0.985	0.895	0.767
J059387	FEMALE	43	212.71	224.93	188270	1215427	929812	0.982	0.885	0.751
J059485	FEMALE	53	140.4	135.35	188270	786572	574830	0.971	0.814	0.645
J059517	FEMALE	50	121.28	133.86	188270	691555	508192	0.948	0.756	0.544
J059519	FEMALE	53	199.06	219.74	188270	1155629	808254	0.91	0.833	0.705
J059595	FEMALE	46	95.32	110.14	188270	547098	386022	0.864	0.687	0.446
J059619	MALE	42	110.09	118.91	188270	630684	444830	0.924	0.725	0.492
J059627	MALE	44	151.62	181.02	188270	862572	633426	0.972	0.783	0.602
J074289	MALE	47	355.62	386.21	188270	2047163	1513338	0.988	0.956	0.88
J074419	MALE	29	230.2	290.26	188270	1333039	998084	0.975	0.865	0.74
J074426	FEMALE	30	273.54	325.39	188270	1582102	1176572	0.982	0.896	0.804
J074497	MALE	22	323.85	383.06	188270	1863364	1379322	0.988	0.954	0.859
J076578	FEMALE	47	181.65	184.66	188270	1039817	727150	0.973	0.845	0.71
J076666	MALE	55	209.79	171.7	188270	1197671	757134	0.987	0.93	0.829
J076676	MALE	44	88.94	89.23	188270	510517	373776	0.915	0.65	0.381
J076746	MALE	28	92.63	121.67	188270	527312	375142	0.911	0.607	0.387
J076845	MALE	47	129.26	139.21	188270	744943	547480	0.97	0.767	0.546
J076916	MALE	46	131.31	190.92	188270	761462	429120	0.962	0.774	0.584
J076917	FEMALE	45	298.99	268.44	188270	1693518	1159306	0.988	0.94	0.871
J076919	FEMALE	25	106.41	114.19	188270	616649	383976	0.93	0.708	0.471
J076948	FEMALE	46	271.99	225.77	188270	1596222	884698	0.982	0.952	0.901
J077106	FEMALE	25	102.27	116.64	188270	581301	390280	0.91	0.668	0.401
J077116	FEMALE	53	360.3	367.94	188270	1997342	1422994	0.962	0.912	0.886
J142917	FEMALE	49	114.53	92.52	188270	643210	457644	0.957	0.792	0.6
J354666	MALE	47	125.65	139.82	188270	721539	533518	0.957	0.758	0.55
J367166	FEMALE	45	96.06	110.42	188270	560616	380980	0.926	0.649	0.429
J400249	MALE	29	597.53	835.1	188270	3378347	2541006	0.935	0.921	0.862
J400287	FEMALE	29	251.2	240.17	188270	1402879	1053626	0.966	0.885	0.798
J406569	MALE	27	377.99	569.49	188270	2175103	1655556	0.982	0.896	0.822
J459495	FEMALE	28	309.66	330.51	188270	1785466	1308166	0.987	0.932	0.849
J459585	MALE	44	331.08	351.83	188270	1903073	1396444	0.987	0.956	0.871
J464659	FEMALE	29	176.84	193.84	188270	1013793	725608	0.981	0.888	0.743
J514895	MALE	44	95.17	109.45	188270	555943	397224	0.903	0.654	0.422
J521638	FEMALE	43	322.8	299.97	188270	1775690	1336476	0.988	0.975	0.922
J521945	MALE	43	135.54	152.83	188270	774703	557078	0.957	0.788	0.616
J522018	MALE	47	139.95	135.79	188270	799212	582404	0.926	0.782	0.629
J522127	FEMALE	47	151.1	159.69	188270	871677	613130	0.967	0.807	0.62
J522339	MALE	41	259.28	419.73	188270	1490662	1132114	0.977	0.857	0.73
J522385	MALE	45	311.8	352.15	188270	1740606	1300928	0.987	0.937	0.851
J522487	FEMALE	46	128.64	140.64	188270	735483	537346	0.965	0.782	0.56
J541488	MALE	43	121.29	102.48	188270	683062	525000	0.954	0.815	0.62
J541526	MALE	46	224.36	209.22	188270	1262637	960294	0.981	0.924	0.823
J541536	MALE	25	255.05	223.2	188270	1437427	1069108	0.988	0.962	0.887
J541558	FEMALE	25	181.33	150.21	188270	1017068	763528	0.961	0.892	0.765
J541565	FEMALE	23	228.12	151.8	188270	1274522	936818	0.969	0.953	0.898
J551578	FEMALE	24	101.23	94.89	188270	578210	375370	0.946	0.674	0.451
J585847	MALE	29	223.47	190.86	188270	1246021	940916	0.965	0.927	0.819
J585856	FEMALE	29	185.13	204.58	188270	1034008	810686	0.94	0.839	0.7
J585857	FEMALE	22	70.54	69.52	188270	409557	300624	0.89	0.519	0.276
J610128	FEMALE	30	308.1	415.42	188270	1725099	1260476	0.982	0.917	0.841
J630499	FEMALE	45	289.28	354.4	188270	1612618	1206756	0.987	0.929	0.802
J638508	MALE	43	297.01	368.7	188270	1690770	1301498	0.986	0.93	0.822
J647999	MALE	45	260.17	294.93	188270	1512465	1101312	0.984	0.917	0.79
J722998	FEMALE	23	253.79	211.29	188270	1438530	945380	0.988	0.959	0.884
J757035	FEMALE	29	280.51	326.7	188270	1627987	1204306	0.985	0.928	0.835
J773046	MALE	49	194.34	279.42	188270	1150747	827198	0.935	0.785	0.563
J781317	FEMALE	43	105.91	99.11	188270	609120	370302	0.93	0.719	0.464
J785649	MALE	30	246.34	269.63	188270	1429426	1037722	0.985	0.91	0.818
J800198	MALE	30	310.35	244.18	188270	1747188	1320938	0.988	0.969	0.918

Sample ID	Gender	BMI (kg/m2)	Avg. Coverage	Std Dev.	Base Pairs	Mapped Reads	Mapped in pairs	Fraction of Bases > 20x	Fraction of Bases > 50x	Fraction of Bases > 80x
J800276	MALE	29	369.44	308.13	188270	2048720	1580944	0.989	0.976	0.949
J843226	MALE	42	108.62	127.98	188270	630989	433700	0.941	0.695	0.474
J843227	FEMALE	49	239.54	262.48	188270	1360712	904678	0.979	0.895	0.802
J859665	FEMALE	45	403.79	464.6	188270	2327763	1736822	0.988	0.971	0.919
J862416	MALE	28	352.97	381.62	188270	2036030	1508286	0.988	0.968	0.91
J894758	FEMALE	30	99.63	121.63	188270	576838	360122	0.927	0.684	0.445
J894759	FEMALE	47	97.35	88.64	188270	563174	357998	0.928	0.724	0.446
J918047	MALE	54	162.69	202.6	188270	967755	690248	0.906	0.746	0.553
J918119	MALE	57	158.73	177.97	188270	907095	648808	0.973	0.839	0.662
J931779	MALE	48	116.88	105.92	188270	666051	450908	0.947	0.791	0.576
J952588	MALE	28	247.71	250.65	188270	1375860	1048286	0.983	0.925	0.824
K013675	MALE	28	164.65	175.2	188270	945244	694140	0.976	0.828	0.673
K013676	MALE	25	105.29	103.41	188270	606681	443354	0.932	0.717	0.469
K157145	FEMALE	26	214	229.6	188270	1202641	911586	0.974	0.872	0.754
K157147	FEMALE	43	420.19	560.04	188270	2363787	1781310	0.988	0.962	0.899
K158519	FEMALE	45	90.92	91.9	188270	522095	376116	0.907	0.649	0.411
K160949	MALE	49	425.98	497.97	188270	2415001	1408488	0.988	0.966	0.891
K195296	FEMALE	29	98.06	88.96	188270	561183	364388	0.893	0.699	0.465
K220606	MALE	26	209.83	200.08	188270	1189102	832996	0.983	0.913	0.799
K221707	MALE	48	624.38	744.99	188270	3517928	1989622	0.988	0.957	0.926
K221708	MALE	48	127.5	190.51	188270	760008	542972	0.876	0.607	0.382
K240326	MALE	50	127.95	138.43	188270	736177	526388	0.896	0.754	0.585
K259639	MALE	44	107.78	100.98	188270	617067	455298	0.958	0.734	0.49
K262497	FEMALE	55	266.95	392.9	188270	1491114	724394	0.952	0.828	0.741
K272206	FEMALE	48	438.13	490.45	188270	2489772	1350084	0.987	0.96	0.889
K272209	MALE	53	199	279.89	188270	1114675	647442	0.973	0.813	0.657
K272216	MALE	43	295.41	346.94	188270	1696418	947300	0.982	0.9	0.824
K276318	MALE	43	89.87	78.58	188270	514126	331202	0.908	0.672	0.424
K304588	MALE	25	200.47	249.68	188270	1144431	477352	0.966	0.821	0.672
K340568	MALE	48	169.26	161.08	188270	949472	690844	0.981	0.85	0.743
K381097	FEMALE	53	73.56	82.38	188270	440638	296626	0.901	0.54	0.284
K381165	FEMALE	49	70.98	62.03	188270	404593	282088	0.902	0.557	0.287
K428998	MALE	42	153.42	157.91	188270	874942	638366	0.976	0.842	0.683
K444817	FEMALE	45	338.94	401.61	188270	1997522	1059486	0.988	0.954	0.889
K456797	MALE	27	241.57	270.02	188270	1419381	1017094	0.975	0.872	0.763
K463728	FEMALE	29	187.36	192.58	188270	1042777	797972	0.975	0.859	0.709
K467599	FEMALE	50	133.33	180.9	188270	740001	216108	0.907	0.72	0.512
K475557	MALE	29	215.31	229.19	188270	1255832	902096	0.984	0.911	0.768
K483309	MALE	27	275.88	396.85	188270	1586795	819282	0.955	0.839	0.758
K511839	FEMALE	30	90.78	87.4	188270	520980	322928	0.907	0.664	0.383
K511849	FEMALE	48	121.56	116.26	188270	699814	451360	0.958	0.801	0.588
K511857	FEMALE	28	113.42	100.75	188270	647545	457232	0.941	0.759	0.548
K518287	FEMALE	25	259.88	332.19	188270	1495579	811452	0.984	0.865	0.751
K555986	FEMALE	28	180.09	185.26	188270	1028318	687626	0.98	0.877	0.729
K556065	FEMALE	46	164.62	251.93	188270	950842	599888	0.976	0.833	0.678
K561809	MALE	27	115.85	121.54	188270	672786	451826	0.947	0.745	0.531
K599688	MALE	24	108.14	105.67	188270	621716	391364	0.931	0.75	0.521
K620537	FEMALE	47	255.55	317.36	188270	1382686	613296	0.983	0.871	0.778
K636646	MALE	27	129.09	120.78	188270	749524	542186	0.974	0.812	0.6
K636688	FEMALE	51	146.73	135.02	188270	835711	323570	0.978	0.853	0.662
K687786	FEMALE	55	144.14	148.11	188270	831333	598360	0.973	0.822	0.668
K687788	FEMALE	55	127.91	148.69	188270	732542	539654	0.937	0.771	0.577
K696106	MALE	26	247.44	332.13	188270	1431658	825390	0.986	0.943	0.839
K696138	FEMALE	30	214.76	215.78	188270	1218125	892882	0.978	0.89	0.767
K728096	FEMALE	24	193.45	242.1	188270	1125640	642450	0.957	0.808	0.65
K736459	FEMALE	25	406.18	512.23	188270	2299519	1190452	0.988	0.955	0.895
K758117	FEMALE	53	207.46	275.08	188270	1182031	466054	0.974	0.817	0.668
K769747	MALE	26	219.43	283.07	188270	1200321	442982	0.931	0.803	0.702
K774666	FEMALE	45	229.53	284.73	188270	1256554	503938	0.978	0.853	0.741
K774668	FEMALE	29	205.26	255.19	188270	1124795	510912	0.975	0.836	0.695
K788548	MALE	50	112	96.07	188270	635818	428210	0.932	0.783	0.552
K799766	MALE	45	193.2	222.93	188270	1095818	832520	0.978	0.888	0.752
K806376	FEMALE	47	108.52	91.64	188270	619048	420826	0.948	0.767	0.522
K806627	FEMALE	51	137.68	216.01	188270	827517	562068	0.967	0.807	0.621
K846997	MALE	51	256.85	307.07	188270	1488802	757820	0.985	0.89	0.771
K881816	FEMALE	51	153.15	157.08	188270	849179	301168	0.974	0.834	0.658

Sample ID	Gender	BMI (kg/m2)	Avg. Coverage	Std Dev.	Base Pairs	Mapped Reads	Mapped in pairs	Fraction of Bases > 20x	Fraction of Bases > 50x	Fraction of Bases > 80x
K902117	MALE	43	99.1	93.2	188270	562005	403960	0.916	0.703	0.484
K911616	MALE	29	104.35	105.95	188270	597988	393780	0.93	0.724	0.457
K918626	FEMALE	52	153.56	174.52	188270	885674	626848	0.967	0.812	0.635
K924395	FEMALE	24	139.36	131.95	188270	781091	575688	0.964	0.841	0.638
K924505	FEMALE	29	187.03	168.49	188270	1057516	768504	0.984	0.9	0.795
K954586	FEMALE	23	148.82	169.84	188270	892542	544294	0.967	0.83	0.667
K954598	FEMALE	27	88.16	91.92	188270	510908	323844	0.896	0.611	0.374
K955127	MALE	42	177.58	208.22	188270	1016907	724714	0.968	0.824	0.661
L027338	FEMALE	47	66.6	55.79	188270	380095	256210	0.927	0.513	0.262
L037608	MALE	45	208.85	289	188270	1202820	495606	0.977	0.827	0.687
L037615	FEMALE	28	148.34	182.31	188270	827138	248056	0.948	0.754	0.547
L037618	FEMALE	44	207.76	221.48	188270	1124253	413850	0.979	0.856	0.757
L037646	MALE	28	253.82	302.84	188270	1430189	568614	0.985	0.894	0.791
L068857	MALE	27	166.51	216.99	188270	913053	437306	0.952	0.744	0.563
L078347	MALE	50	84.51	88.82	188270	452015	0	0.902	0.591	0.349
L078378	MALE	27	247.98	216.1	188270	1393252	901322	0.989	0.974	0.891
L078389	MALE	27	122.81	125.32	188270	701256	521502	0.955	0.762	0.54
L095676	MALE	26	120.81	110.1	188270	680802	505128	0.946	0.78	0.572
L155378	FEMALE	24	100.47	132.27	188270	578233	401676	0.91	0.679	0.455
L155386	MALE	29	144.96	162.88	188270	833670	590208	0.96	0.8	0.608
L157068	FEMALE	27	197.03	250.35	188270	1116301	480848	0.971	0.82	0.665
L205849	MALE	46	305.12	307.72	188270	1690191	903212	0.989	0.947	0.862
L211127	FEMALE	27	138.5	151.34	188270	797171	549582	0.963	0.779	0.593
L211128	FEMALE	51	154.39	182.98	188270	844489	404768	0.956	0.77	0.558
L211438	MALE	27	366.8	492.09	188270	2052735	1351400	0.986	0.91	0.779
L229228	FEMALE	50	181.3	218.05	188270	987515	534186	0.949	0.81	0.646
L253786	FEMALE	24	181.42	244.66	188270	996245	476586	0.946	0.763	0.579
L253787	FEMALE	50	228.14	321.59	188270	1289933	960032	0.963	0.826	0.691
L255016	FEMALE	45	189.48	213.93	188270	1027023	468480	0.975	0.843	0.673
L255376	FEMALE	29	196.77	193.37	188270	1118315	851914	0.977	0.894	0.771
L273097	MALE	49	1682.66	1512.92	188270	9384531	5713718	0.991	0.99	0.99
L302476	FEMALE	30	180.23	165.84	188270	1024758	753276	0.978	0.883	0.747
L311079	MALE	29	249.71	314.29	188270	1362847	600742	0.978	0.861	0.741
L311086	FEMALE	26	225.07	296.92	188270	1237860	588558	0.971	0.825	0.7
L311088	MALE	27	225.27	297.76	188270	1244844	718312	0.975	0.826	0.683
L317787	MALE	45	99.63	94.2	188270	569778	383890	0.959	0.699	0.445
L342769	FEMALE	44	218.78	222.34	188270	1219994	921450	0.931	0.905	0.828
L342778	FEMALE	26	99.45	102.53	188270	572393	386964	0.905	0.678	0.436
L357649	FEMALE	27	531.62	782.48	188270	3015520	2240308	0.988	0.957	0.862
L357679	MALE	42	122.55	168.26	188270	711485	499372	0.942	0.713	0.509
L361556	MALE	28	104.91	97.82	188270	603687	424422	0.931	0.701	0.492
L426546	FEMALE	48	274.56	347.21	188270	1611951	1159886	0.979	0.868	0.788
L429245	MALE	48	209.51	202.5	188270	1196897	765332	0.98	0.901	0.769
L454716	MALE	45	74.87	85.97	188270	429740	323998	0.853	0.528	0.286
L461017	MALE	27	230.39	226.02	188270	1275580	1010734	0.986	0.922	0.795
L532318	MALE	52	272.2	317.5	188270	1603852	1167642	0.983	0.898	0.794
L564275	MALE	27	104.15	111.93	188270	598847	367506	0.939	0.714	0.457
L607938	FEMALE	28	81.32	81.5	188270	469712	292772	0.9	0.598	0.335
L619216	FEMALE	28	119.26	119.36	188270	668418	503178	0.951	0.734	0.514
L620249	MALE	29	133.34	158.51	188270	779522	572606	0.9	0.726	0.513
L678458	MALE	52	178.59	168.04	188270	1009260	751894	0.984	0.901	0.753
L679178	FEMALE	49	203.94	220.72	188270	1160449	848584	0.975	0.881	0.763
L703858	FEMALE	25	189.86	202.59	188270	1080300	791362	0.975	0.863	0.735
L711309	FEMALE	28	270.79	215.96	188270	1439987	342	0.988	0.962	0.905
L739505	MALE	54	173.68	221.37	188270	1009484	752598	0.961	0.802	0.612
L741529	FEMALE	46	162.98	201.1	188270	932259	704506	0.982	0.877	0.69
L823625	MALE	43	117.29	117.11	188270	673452	408844	0.945	0.75	0.539
L836327	FEMALE	26	348.51	334.24	188270	1938629	971990	0.989	0.97	0.918
L836436	FEMALE	25	202.05	204.29	188270	1148710	835830	0.983	0.903	0.778
L836437	MALE	25	147.03	177.6	188270	843972	602108	0.961	0.792	0.615
L836438	MALE	27	216.49	215.89	188270	1223971	901300	0.936	0.869	0.79
L845058	MALE	49	156.3	207.8	188270	890885	676052	0.963	0.823	0.64
L887325	FEMALE	58	363.97	326.22	188270	2054476	1407412	0.989	0.971	0.926
L927807	MALE	43	132.24	161.36	188270	765685	529304	0.954	0.741	0.557
L966268	MALE	26	149.07	196.22	188270	844128	626950	0.949	0.725	0.536
L979627	MALE	27	285.72	309.23	188270	1585335	1229676	0.983	0.906	0.796

Sample ID	Gender	BMI (kg/m2)	Avg. Coverage	Std Dev.	Base Pairs	Mapped Reads	Mapped in pairs	Fraction of Bases > 20x	Fraction of Bases > 50x	Fraction of Bases > 80x
L980967	MALE	44	300.71	341	188270	1695306	1260604	0.987	0.919	0.833
L981495	MALE	54	306.11	325.72	188270	1725900	1296384	0.987	0.92	0.834
L997999	FEMALE	42	107.4	102.88	188270	631575	437044	0.953	0.751	0.518
M028096	MALE	29	271.51	338.05	188270	1510943	1154740	0.983	0.911	0.796
M137156	MALE	26	176.56	170.55	188270	1005519	717704	0.975	0.872	0.719
M137166	MALE	28	277.76	346.8	188270	1596166	998502	0.988	0.961	0.886
M137167	MALE	29	177.56	166.27	188270	1009529	737270	0.977	0.879	0.731
M137169	MALE	29	111.55	267.48	188270	647255	455630	0.919	0.654	0.44
M158308	MALE	56	151.91	144.91	188270	878165	628814	0.981	0.838	0.672
M158317	MALE	53	96.48	135.2	188270	557875	402900	0.912	0.638	0.415
M160478	FEMALE	30	273.49	261.57	188270	1534994	1063282	0.988	0.959	0.861
M189706	FEMALE	28	158.92	145.51	188270	896740	615954	0.959	0.816	0.702
M215109	FEMALE	27	141.23	130.7	188270	846308	472102	0.964	0.854	0.68
M295506	MALE	46	286.54	329.14	188270	1663946	1248304	0.986	0.929	0.838
M297429	FEMALE	42	271.77	255.1	188270	1561595	1168074	0.986	0.946	0.861
M332096	FEMALE	30	260.89	316.76	188270	1528182	1104090	0.982	0.881	0.772
M501609	MALE	29	173.64	148.17	188270	983306	731638	0.978	0.888	0.746
M503169	FEMALE	27	231.47	278.87	188270	1297394	960540	0.974	0.859	0.756
M518406	FEMALE	24	268.92	294.88	188270	1566095	1134516	0.987	0.925	0.825
M584176	FEMALE	42	238.64	254.43	188270	1333810	1014914	0.961	0.871	0.796
M592697	MALE	29	202.38	188.1	188270	1164085	810692	0.98	0.917	0.795
M646718	MALE	27	185.04	221.1	188270	1080036	795494	0.976	0.815	0.647
M689547	FEMALE	47	307.42	314.56	188270	1783615	1325724	0.986	0.944	0.855
M706097	FEMALE	49	133.66	129.49	188270	757061	559006	0.958	0.791	0.624
M738507	MALE	26	530.15	658.35	188270	3001554	2229118	0.988	0.974	0.919
M738509	FEMALE	27	234.08	275.09	188270	1327674	994122	0.981	0.89	0.76
M761688	MALE	44	64.58	58.5	188270	371849	239720	0.855	0.5	0.241
M761689	FEMALE	28	106.22	98.75	188270	599742	408486	0.955	0.739	0.501
M769288	FEMALE	23	187.15	179.76	188270	1064729	765110	0.974	0.857	0.743
M784767	FEMALE	24	131.69	139.18	188270	747283	556344	0.946	0.75	0.579
M851785	MALE	42	636.19	869.13	188270	3588594	2694960	0.989	0.966	0.892
M865287	FEMALE	27	97.23	88.17	188270	551127	366424	0.95	0.698	0.432
M889206	MALE	48	97.1	82.4	188270	549810	374664	0.962	0.729	0.46
M920876	MALE	43	163.81	144.09	188270	932314	688146	0.962	0.862	0.734
M937689	MALE	52	98.04	113.06	188270	560611	408340	0.924	0.681	0.477
N015046	FEMALE	26	167.16	139.42	188270	948532	690364	0.978	0.873	0.74
N052156	MALE	42	169.5	178.69	188270	955657	736154	0.97	0.845	0.687
N053108	MALE	51	136.61	143.2	188270	783253	566414	0.902	0.727	0.579
N060369	FEMALE	25	185.34	187.91	188270	1061542	759702	0.977	0.873	0.725
N094585	MALE	28	136	136.96	188270	767219	594184	0.962	0.799	0.608
N098465	MALE	25	244.68	319.69	188270	1370328	1068776	0.974	0.852	0.702
N110696	MALE	28	264.57	304.8	188270	1558891	1104052	0.979	0.897	0.805
N132108	MALE	28	169.74	145.44	188270	963033	710876	0.963	0.884	0.729
N137945	MALE	43	102.81	125.95	188270	603508	420970	0.952	0.721	0.473
N162606	MALE	46	287.16	318.89	188270	1606716	1214342	0.987	0.943	0.832
N183339	MALE	47	120.9	118.95	188270	692879	477584	0.915	0.737	0.564
N229556	FEMALE	28	219.28	187	188270	1239731	772338	0.988	0.952	0.845
N303116	MALE	29	249.5	273.5	188270	1436551	1072422	0.979	0.903	0.816
N323976	FEMALE	27	261.98	213.81	188270	1493186	918690	0.988	0.967	0.904
N362738	FEMALE	48	294.53	222.78	188270	1643633	1235886	0.98	0.958	0.929
N362889	MALE	29	247.93	277.63	188270	1407700	1044416	0.982	0.902	0.798
N550499	MALE	42	298.35	328.45	188270	1685833	1238462	0.985	0.922	0.838
N550508	MALE	27	187.86	208	188270	1105147	629832	0.982	0.92	0.783
N552338	MALE	29	268.47	304.15	188270	1510016	1147204	0.987	0.929	0.815
N594787	FEMALE	51	315.17	349.28	188270	1830633	1347454	0.985	0.918	0.831
N680387	FEMALE	49	334.46	363.02	188270	1947573	1382494	0.988	0.953	0.868
N725817	FEMALE	28	113.66	105.44	188270	647081	477128	0.937	0.735	0.553
N741249	MALE	28	247.75	274.95	188270	1413291	1072046	0.983	0.915	0.797
N763157	MALE	29	608.45	823.6	188270	3457524	2549818	0.99	0.977	0.922
N898767	MALE	28	130.97	131.33	188270	743314	546566	0.958	0.753	0.556
N924728	MALE	28	168.25	145.16	188270	950052	685592	0.981	0.88	0.744
N945607	MALE	28	185.9	213.99	188270	1058607	767058	0.972	0.826	0.718
N945619	FEMALE	29	125.04	142.82	188270	703188	518628	0.954	0.756	0.543
N948286	FEMALE	47	105.66	105.1	188270	599916	450172	0.94	0.728	0.489
N976086	FEMALE	54	287.88	313.9	188270	1643469	1244438	0.986	0.937	0.84
O070248	MALE	54	289.4	322.09	188270	1619257	1212260	0.984	0.922	0.821

Sample ID	Gender	BMI (kg/m2)	Avg. Coverage	Std Dev.	Base Pairs	Mapped Reads	Mapped in pairs	Fraction of Bases > 20x	Fraction of Bases > 50x	Fraction of Bases > 80x
O070250	FEMALE	44	293.76	322.31	188270	1642387	1236884	0.985	0.919	0.823
O162106	MALE	29	163.49	146.58	188270	920074	696520	0.958	0.852	0.696
O184815	FEMALE	43	271.54	324.4	188270	1516605	1156934	0.98	0.885	0.79
O198108	FEMALE	47	131.23	271.02	188270	743812	556016	0.94	0.73	0.554
O198115	FEMALE	42	144.41	180.37	188270	813409	613794	0.951	0.804	0.638
O225807	FEMALE	25	195.62	199.42	188270	1106973	822578	0.983	0.903	0.755
O251735	MALE	51	91.53	73.67	188270	517970	352966	0.925	0.724	0.45
O264628	FEMALE	44	329.03	343.94	188270	1896587	1411128	0.988	0.954	0.876
O276207	FEMALE	53	290.58	312.09	188270	1629703	1233796	0.986	0.935	0.85
O283876	MALE	28	144.37	128.53	188270	811229	618474	0.959	0.843	0.667
O298496	FEMALE	23	277.78	330.29	188270	1597777	1204492	0.984	0.907	0.791
O435338	MALE	29	140.87	117.83	188270	792032	597548	0.958	0.83	0.65
O436587	FEMALE	27	205.5	224.06	188270	1161113	857886	0.976	0.86	0.729
O436588	MALE	29	125.24	137.93	188270	710535	523874	0.94	0.722	0.497
O480168	MALE	27	198.67	191.98	188270	1119927	834906	0.981	0.888	0.765
O547755	MALE	42	221.18	248.34	188270	1240915	936672	0.979	0.855	0.736
O635009	FEMALE	49	342.7	394.15	188270	2002216	1443680	0.988	0.953	0.872
O674577	MALE	28	153.92	141.8	188270	872095	634464	0.971	0.845	0.694
O761327	MALE	25	278.97	362.03	188270	1638263	1173004	0.984	0.894	0.784
O803929	FEMALE	44	291.08	413.08	188270	1665227	1219356	0.961	0.815	0.69
O815265	FEMALE	25	175.42	165.75	188270	997151	722968	0.972	0.862	0.736
O825675	FEMALE	22	244.56	308.57	188270	1380908	1033840	0.981	0.875	0.753
O831206	MALE	24	498.53	649.03	188270	2843317	2076020	0.937	0.924	0.875
O841936	MALE	44	260.7	279.31	188270	1533610	1089106	0.987	0.941	0.843
O962259	FEMALE	49	129.28	127.4	188270	724019	545066	0.947	0.792	0.576
O995536	FEMALE	25	237.73	282.78	188270	1389842	993992	0.973	0.869	0.77
P011849	MALE	47	158.84	162.04	188270	892966	655366	0.979	0.81	0.679
P907247	MALE	26	163.05	160.41	188270	933203	669836	0.974	0.831	0.657
P907259	MALE	28	187.73	179.45	188270	1064892	794172	0.979	0.873	0.741
P943329	FEMALE	28	239.29	221.04	188270	1341180	1004070	0.986	0.948	0.845

Supplemental Table 2: List of potential tri-allelic SNVs in the two genes: Tri-allelic variant calls are likely to be sequencing errors. Out of the 15 identified, only 5 (33%) had sufficient quality and coverage to be true tri-allelic variants. Four out of 5 are private variants and observed only once (MAF=0.002) and one is observed three times (Supplemental Table 2). This small number of tri-allelic variants (0.34% of the 1448 SNVs) is consistent with the proportion of tri-allelic SNPs in the Seattle SNPs database which contains 67 tri-allelic SNPs (0.224%) (Huebner et al. 2007). Sequencing represents the most direct method of detecting and genotyping tri-allelic variants, which can be miscalled by standard genotyping platforms.

			allelic counts					
**chr**	**Coordinates**	**RefBase**	**A**	**C**	**G**	**T**	**comments**	**tri-allelic**
1	46634868	G	0	13	514	17	common indel at same location	-
1	46642725	G	3	0	572	1	high error rate in tail of reads	-
3	128912259	G	1	0	576	1	high error rate in tail of reads	-
3	128923399	C	118	443	0	1	4bp common indel at same location	-
3	128994239	G	3	1	558	0	GA genotype is incorrect	-
3	129024117	T	58	8	10	30	low coverage	-
3	129024128	A	10	4	2	4	low coverage	-
3	129025497	T	0	290	4	10	low coverage	-
3	129025993	C	150	184	24	140	low coverage	-
3	129025994	A	2	252	240	2	low coverage	-
3	128912257	C	0	411	164	1	one individual with CT genotype	yes
3	128918936	C	0	558	3	15	3 individuals with CG genotype	yes
3	128931676	G	260	2	316	0	one individual with CC genotype	yes
3	129026285	G	1	5	568	0	one individual with GA genotype	yes
3	129030212	C	7	568	0	1	one individual with CT genotype	yes

Supplemenal Table 3 : List of SNPs having published associations with obesity, and the p-value of their association in the 289 CRESCENDO samples

Associated SNP	Gene	Reference	p-value*
rs10485170	CNR1	18375449	0.554
rs1049353	CNR1	17873324	0.984
rs12720071	CNR1	17405839	0.606
rs2023239	CNR1	18375449	0.472
rs6454674	CNR1	18375449	0.253
rs806381	CNR1	18375449	0.280
rs16986921	CTNNBL1	18325910	0.896
rs6013029	CTNNBL1	18325910	0.899
rs6020712	CTNNBL1	18325910	0.841
rs1121980	FTO	18159244	**0.018**
rs1421085	FTO	17496892	**0.020**
rs17817449	FTO	17496892	**0.029**
rs3751812	FTO	18335027	**0.026**
rs9930506	FTO	17658951	**0.021**
rs9939609	FTO	17434869	**0.030**
rs7566605	INSIG2	17137505	0.925
rs12970134	MC4R	18454146	0.652
rs17782313	MC4R	18454148	0.488
rs477181	MC4R	19073769	0.788
rs502933	MC4R	19073769	0.830
rs6602024	PFKP	17658951	0.962

* in bold p-value<0.05

Supplemental Table 4 : List of the 31 significant locus-variantd identfied by RareCover. The three locus-variants mentioned in the text are highlighted in bold

First SNV in Locus-Variant	Interval	position	-Log10(p-value)	RareCover Selected SNVs	Selected SNVs
chr1_46624885	FAAH Promoter	46624885	2.04	17	chr1_46626821, chr1_46627662, chr1_46627232, chr1_46625037, chr1_46629717, chr1_46627175, chr1_46626052, chr1_46626861, chr1_46626422, chr1_46629129, chr1_46628662, chr1_46628583, chr1_46625516, chr1_46629431, chr1_46626376, chr1_46628062, chr1_46627269
chr1_46625037	FAAH Promoter	46625037	2.08	17	chr1_46626821, chr1_46627662, chr1_46627232, chr1_46625037, chr1_46629717, chr1_46627175, chr1_46626052, chr1_46626861, chr1_46626422, chr1_46629129, chr1_46628662, chr1_46628583, chr1_46625516, chr1_46629431, chr1_46626376, chr1_46628062, chr1_46627269
chr1_46626376	FAAH Promoter	46626376	2.53	16	chr1_46626821, chr1_46630633, chr1_46627232, chr1_46629717, chr1_46627175, chr1_46631328, chr1_46626861, chr1_46626422, chr1_46629129, chr1_46628662, chr1_46628583, chr1_46630187, chr1_46629431, chr1_46626376, chr1_46628062, chr1_46627269
chr1_46626422	FAAH Promoter	46626422	2.09	15	chr1_46626821, chr1_46630633, chr1_46627232, chr1_46629717, chr1_46627175, chr1_46631328, chr1_46626861, chr1_46626422, chr1_46629129, chr1_46628662, chr1_46628583, chr1_46630187, chr1_46629431, chr1_46628062, chr1_46627269
chr1_46626456	FAAH Promoter	46626456	2.20	14	chr1_46626821, chr1_46630633, chr1_46627232, chr1_46629717, chr1_46627175, chr1_46631328, chr1_46626861, chr1_46629129, chr1_46628662, chr1_46628583, chr1_46630187, chr1_46629431, chr1_46628062, chr1_46627269
chr1_46626821	FAAH Promoter	46626821	2.58	16	chr1_46626821, chr1_46630633, chr1_46627232, chr1_46629717, chr1_46627175, chr1_46631328, chr1_46626861, chr1_46629129, chr1_46628662, chr1_46628583, chr1_46631810, chr1_46630187, chr1_46629431, chr1_46628062, chr1_46631519, chr1_46627269
**chr1_46626861**	FAAH Promoter	**46626861**	**2.65**	**15**	**chr1_46630633, chr1_46627232, chr1_46629717, chr1_46627175, chr1_46631328, chr1_46626861, chr1_46629129, chr1_46628662, chr1_46628583, chr1_46631810, chr1_46630187, chr1_46629431, chr1_46628062, chr1_46631519, chr1_46627269**
chr1_46627175	FAAH Promoter	46627175	2.33	14	chr1_46630633, chr1_46627232, chr1_46629717, chr1_46627175, chr1_46631328, chr1_46629129, chr1_46628662, chr1_46628583, chr1_46631810, chr1_46630187, chr1_46629431, chr1_46628062, chr1_46631519, chr1_46627269
chr1_46627232	FAAH Promoter	46627232	2.42	13	chr1_46630633, chr1_46627232, chr1_46629717, chr1_46631328, chr1_46629129, chr1_46628662, chr1_46628583, chr1_46631810, chr1_46630187, chr1_46629431, chr1_46628062, chr1_46631519, chr1_46627269
chr1_46627269	FAAH Promoter	46627269	2.08	12	chr1_46630633, chr1_46629717, chr1_46631328, chr1_46629129, chr1_46628662, chr1_46628583, chr1_46631810, chr1_46630187, chr1_46629431, chr1_46628062, chr1_46631519, chr1_46627269
chr1_46627603	FAAH Promoter	46627603	2.00	12	chr1_46630633, chr1_46629717, chr1_46631328, chr1_46629129, chr1_46628662, chr1_46628583, chr1_46631810, chr1_46630187, chr1_46629431, chr1_46632268, chr1_46628062, chr1_46631519
chr1_46627662	FAAH Promoter	46627662	2.09	12	chr1_46630633, chr1_46629717, chr1_46631328, chr1_46629129, chr1_46628662, chr1_46628583, chr1_46631810, chr1_46630187, chr1_46629431, chr1_46632268, chr1_46628062, chr1_46631519
chr1_46628062	FAAH Promoter	46628062	2.05	13	chr1_46630633, chr1_46629717, chr1_46631328, chr1_46629129, chr1_46628662, chr1_46628583, chr1_46631810, chr1_46632771, chr1_46630187, chr1_46629431, chr1_46632268, chr1_46628062, chr1_46631519
chr1_46628584	FAAH Promoter	46628584	2.10	13	chr1_46630633, chr1_46628584, chr1_46629717, chr1_46631328, chr1_46629129, chr1_46628662, chr1_46631810, chr1_46632771, chr1_46633092, chr1_46630187, chr1_46629431, chr1_46632268, chr1_46631519
chr1_46629068	FAAH Promoter	46629068	2.04	11	chr1_46630633, chr1_46632771, chr1_46629717, chr1_46633092, chr1_46630187, chr1_46631328, chr1_46629431, chr1_46632268, chr1_46629129, chr1_46631810, chr1_46631519
chr1_46629129	FAAH Promoter	46629129	2.10	11	chr1_46630633, chr1_46632771, chr1_46629717, chr1_46633092, chr1_46630187, chr1_46631328, chr1_46629431, chr1_46632268, chr1_46629129, chr1_46631810, chr1_46631519
chr1_46629147	FAAH Promoter	46629147	2.04	10	chr1_46630633, chr1_46632771, chr1_46629717, chr1_46633092, chr1_46630187, chr1_46631328, chr1_46629431, chr1_46632268, chr1_46631810, chr1_46631519

First SNV in Locus-Variant	Interval	position	-Log10(p-value)	RareCover Selected SNVs	Selected SNVs
chr1_46629215	FAAH Promoter	46629215	2.02	10	chr1_46630633, chr1_46632771, chr1_46629717, chr1_46633092, chr1_46630187, chr1_46631328, chr1_46629431, chr1_46632268, chr1_46631810, chr1_46631519
chr1_46629280	FAAH Promoter	46629280	2.13	10	chr1_46630633, chr1_46632771, chr1_46629717, chr1_46633092, chr1_46630187, chr1_46631328, chr1_46629431, chr1_46632268, chr1_46631810, chr1_46631519
chr1_46629431	FAAH Promoter	46629431	2.22	10	chr1_46630633, chr1_46632771, chr1_46629717, chr1_46633092, chr1_46630187, chr1_46631328, chr1_46629431, chr1_46632268, chr1_46631810, chr1_46631519
chr1_46629606	FAAH Promoter	46629606	2.01	9	chr1_46630633, chr1_46632771, chr1_46629717, chr1_46633092, chr1_46630187, chr1_46631328, chr1_46632268, chr1_46631810, chr1_46631519
chr1_46629717	FAAH Promoter	46629717	2.13	9	chr1_46630633, chr1_46632771, chr1_46629717, chr1_46633092, chr1_46630187, chr1_46631328, chr1_46632268, chr1_46631810, chr1_46631519
**chr3_128967692**	MGLL intron 3	**128967692**	**2.02**	**9**	**chr3_128968574, chr3_128971683, chr3_128972166, chr3_128969940, chr3_128968584, chr3_128968631, chr3_128970892, chr3_128969493, chr3_128969236**
chr3_129029491	MGLL Promoter	129029491	2.16	10	chr3_129033308, chr3_129031511, chr3_129031044, chr3_129032662, chr3_129034259, chr3_129031864, chr3_129031107, chr3_129030019, chr3_129034402, chr3_129031590
chr3_129030019	MGLL Promoter	129030019	2.39	11	chr3_129033308, chr3_129031511, chr3_129034757, chr3_129031044, chr3_129032662, chr3_129034259, chr3_129031864, chr3_129031107, chr3_129030019, chr3_129034402, chr3_129031590
chr3_129030091	MGLL Promoter	129030091	2.14	10	chr3_129033308, chr3_129031511, chr3_129034757, chr3_129031044, chr3_129032662, chr3_129034259, chr3_129031864, chr3_129031107, chr3_129034402, chr3_129031590
chr3_129030443	MGLL Promoter	129030443	2.37	10	chr3_129033308, chr3_129031511, chr3_129034757, chr3_129031044, chr3_129032662, chr3_129034259, chr3_129031864, chr3_129031107, chr3_129034402, chr3_129031590
chr3_129030863	MGLL Promoter	129030863	2.40	10	chr3_129033308, chr3_129031511, chr3_129034757, chr3_129031044, chr3_129032662, chr3_129034259, chr3_129031864, chr3_129031107, chr3_129034402, chr3_129031590
**chr3_129030872**	MGLL Promoter	**129030872**	**2.86**	**10**	**chr3_129033308, chr3_129031511, chr3_129034757, chr3_129031044, chr3_129032662, chr3_129034259, chr3_129031864, chr3_129031107, chr3_129034402, chr3_129031590**
chr3_129031044	MGLL Promoter	129031044	2.64	10	chr3_129033308, chr3_129031511, chr3_129034757, chr3_129031044, chr3_129032662, chr3_129034259, chr3_129031864, chr3_129031107, chr3_129034402, chr3_129031590
chr3_129031107	MGLL Promoter	129031107	2.73	9	chr3_129033308, chr3_129031511, chr3_129034757, chr3_129032662, chr3_129034259, chr3_129031864, chr3_129031107, chr3_129034402, chr3_129031590

Supplemental Table 5 : Occurrence in cases and controls of the variants included in the most significant locus-variants in each of the three intervals identified by RareCover algorithm

Interval	NCBI36 Coordinates	minor/major alleles	Number of minor alleles	
			Cases	Controls
FAAH Promoter	chr1_46626861	T/C	1	0
	chr1_46627175	T/C	1	0
	chr1_46627232	T/G	1	0
	chr1_46627269	T/C	1	0
	chr1_46628062	C/G	1	0
	chr1_46628583	C/T	1	0
	chr1_46628662	T/C	1	0
	chr1_46629129	T/C	1	0
	chr1_46629431	A/G	1	0
	chr1_46629717	A/T	4	0
	chr1_46630187	G/T	1	0
	chr1_46630633	C/T	2	0
	chr1_46631328	G/A	4	0
	chr1_46631519	A/G	1	0
	chr1_46631810	A/G	2	0
MGLL intron 3	chr3_128968574	T/C	1	0
	chr3_128968584	C/T	1	0
	chr3_128968631	A/C	2	0
	chr3_128969236	C/T	1	0
	chr3_128969493	G/A	1	0
	chr3_128969940[a]	G/T	14	2
	chr3_128970892	G/A	3	0
	chr3_128971683	A/G	1	0
	chr3_128972166	C/T	1	0
MGLL Promoter	chr3_129033308	A/G	1	0
	chr3_129031511	C/G	1	0
	chr3_129034757	G/A	1	0
	chr3_129031044	T/G	1	0
	chr3_129032662	T/G	1	0
	chr3_129034259	A/G	1	0
	chr3_129031864	A/C	3	0
	chr3_129031107	A/C	16	6
	chr3_129034402	T/C	4	1
	chr3_129031590	G/T	9	2

a- this SNV was identified as signiifcantly associated with high BMI using a single marker test

Supplemental Table 6 : List of primers used in LR-PCR

Name	Sequence	Application	Chr	Amplicon Start	Amplicon End+F90	Amplicon Length
LR_1519929_1	AATGATGTTGATTGTCAGCCTACCTAAGTCTG	LR-PCR	chr3	128994559	128998354	3796
LR_1519929_2	CATCTTTGCTGTGGCTTGTAATTTTCTAGTTG	LR-PCR				
LR_155000_1	ATATGTATTCTCTCCCAACTCAAGATACCC	LR-PCR	chr3	128885889	128892991	7103
LR_155000_2	CCTTTCCTCTAGAGTCACTAATTTTACCTG	LR-PCR				
LR_1550001_1	AGAGGATAATCAAGGATCAGCCTACAATTC	LR-PCR	chr3	128904097	128908255	4159
LR_1550001_2	ACTTACATTCCCCACAGCATATACTTGTCT	LR-PCR				
LR_1550002_1	GAGATTACAGAAGTTCCCAAACAGTGCT	LR-PCR	chr3	128937605	128949807	12203
LR_1550002_2	GATTTAATTCGAATGGTCCTCTGAGTGT	LR-PCR				
LR_1550003_1	ACAAGAGAAACTGTGACTTCCTAGCTC	LR-PCR	chr3	129019374	129024116	4743
LR_1550003_2	GTGATGACATGAACTAGTAGTGGAGAAT	LR-PCR				
LR_1550004_1	CCACCAAAATAGTCTCTGCTTTAAAATATAC	LR-PCR	chr3	128892885	128904210	11326
LR_1550004_2	AGGAGTTCAAACCTTCATACTAAAAAGTAAT	LR-PCR				
LR_1550005_1	AGTGACAGTTTACAGTACATTAAGGAGAGTG	LR-PCR	chr3	128912261	128923122	10862
LR_1550005_2	CTAAACCAGGAAAAAGTATAGTATAGCAATC	LR-PCR				
LR_1550006_1	CTACTAGTTCATGTCATCACTTTTCCTAAAC	LR-PCR*	chr3	129024097	129032789	8693
LR_1550006_2	ATAGTACAGTCTTGCTTTTCCTCTATCATTC	LR-PCR*				
LR_1550007_1	CATTCCTTCTGCTTCTTGGATGTAAAAT	LR-PCR	chr1	46621328	46632948	11621
LR_1550007_2	GAATATCTTAGGGCAAGGAGAGATTCTG	LR-PCR				
LR_1550028_1	AGTCCCTACTGCTGCTCTATCTCCAGTG	LR-PCR	chr3	128880455	128891448	10994
LR_1550028_2	AAAATAAAAACTTTCCTGAAATTGGGACTGT	LR-PCR				
LR_1550029_1	GGTCTATGTTAGCAATTGGTGAAAGAAGAAG	LR-PCR*	chr3	128890768	128901740	10973
LR_1550029_2	AGGTTTCTAAATTTCACAGAGATCACTCAGG	LR-PCR*				
LR_1550030_1	GTTCCATGATGAAATGCAATATCAGAGTTTA	LR-PCR	chr3	128900900	128911910	11011
LR_1550030_2	GTGTTCTAAACATGAAAGGAGCCAACAGT	LR-PCR				
LR_1550031_1	CTAGGAGTTTTCAACCCCTGATCTAGAAAGT	LR-PCR	chr3	128910956	128921931	10976
LR_1550031_2	ACTCTGGCTTGTTGACAAGGTATCAGTTTAG	LR-PCR				
LR_1550032_1	TGACCTACTATCTCACCTCTTTCTCCTTTGA	LR-PCR	chr3	128921723	128932703	10981
LR_1550032_2	GGAGAAAAAGAATTCTCATTACAGATGCAAA	LR-PCR				
LR_1550033_1	ATCCAGTCTCCTTCTTTGCATGTTATATCC	LR-PCR	chr3	128931678	128942677	11000
LR_1550033_2	AACAGAGACCTAGTTGTCCTGTCTCTCTTTG	LR-PCR				
LR_1550034_1	GGATTCTGGACGTCTGTTTTCATACAAG	LR-PCR	chr3	128942353	128953361	11009
LR_1550034_2	CAGATTCTAAAATGAAATTCTCTGAGGGACA	LR-PCR				
LR_1550035_1	CATATCTGCTCTTGAGAAACCACTGTAATTG	LR-PCR	chr3	128952406	128963373	10968
LR_1550035_2	ATATTTAGTGCTTGTCTGGGCATTTGAGAAT	LR-PCR				
LR_1550036_1	AAAAGTTAAAACTGTAGCAAGGTGCAAGTGT	LR-PCR	chr3	128962288	128973287	11000
LR_1550036_2	TGGTAAGTGTGGCACTAAGTCCTAGAATGAT	LR-PCR				
LR_1550037_1	CATTTAGCTTTAAACGTTTTCATCTGCCTTA	LR-PCR	chr3	128973194	128984210	11017
LR_1550037_2	TCTAGCTCTCCGTAGGTCTTCTGCTAAGAAT	LR-PCR				
LR_1550038_1	CATTGTCAAATAAGAGGAGTTTGCTGTTAAA	LR-PCR	chr3	128983864	128994876	11013
LR_1550038_2	CTTATTAGGATTTATGCCAATTGGGGATTTT	LR-PCR				
LR_1550039_1	CTCTGTAACTTGCATGAGTCTAGGGAGAG	LR-PCR	chr3	128994772	129005772	11001
LR_1550039_2	CCATTGATGTTACCTCCTAACTAGCCATCT	LR-PCR				
LR_1550040_1	ACTCATTTATGGGATGACTTTGAATGAAGAT	LR-PCR	chr3	129005066	129016078	11013
LR_1550040_2	GTTTAGATCTTAATCCCAGATGCACTTTGTC	LR-PCR				
LR_1550042_1	AGCCTGTGGTCTAACTTGAGAAATAAAACAA	LR-PCR	chr3	129025997	129037009	11013
LR_1550042_2	CCACTTTTATTTGCGAGCAGAGTCTACTTTA	LR-PCR				
LR_1550045_1	TGATTATCAAATTCAAGATTTCCTGGGTAAG	LR-PCR	chr1	46642728	46653751	11024
LR_1550045_2	AAGGCAACTCTTCTAGAAAGAATCTGAGACA	LR-PCR				
LR_1550047_1	CAGAGCAGATGTGTAACTCTCCAGAATCTC	LR-PCR	chr1	46632900	46642759	9860
LR_1550047_2	CCTTACCCAGGAAATCTTGAATTTGATAAT	LR-PCR				
LR_1550048_1	AAAGAATCTCACGATTGCTGCCTATTG	LR-PCR	chr3	46633060	46637356	4297
LR_1550048_2	GCATAAATAGGTGTTCTGTGATGTCTCTCC	LR-PCR				
LR_1550050_1	AGTCATTCTCAACTGAGGGTTACGCATC	LR-PCR	chr3	128949810	128961030	11221
LR_1550050_2	CTCTTGAGCACGTGATTCAGAAGAGAG	LR-PCR				
LR_1550051_1	CGAAAACATCGGATTTATTAGGATTAGCTG	LR-PCR	chr3	128890602	128901632	11031
LR_1550051_2	GAGAGATCTAAACAATGGAGGAAGAAGGTG	LR-PCR				
LR_422633_1	ATATTATCTTCCTACATTGGGCCTCGGCTTTG	LR-PCR	chr3	128997820	129005351	7532
LR_422633_2	TGGAATGGGATGAACGAGGGAATGAGTAA	LR-PCR				
LR_422638_1	GTTGATCTGTGTGATTCTGCACCTGCCGTGTA	LR-PCR	chr3	128907784	128912599	4816

Name	Sequence	Application	Chr	Amplicon Start	Amplicon End+F90	Amplicon Length
LR_422638_2	CACTGCCAAGGCCCTACTATGTGCATGTCC	LR-PCR	chr3	128907784	128912599	4816
LR_422641_1	CAATTCATCCATCCCGACAGGCTTAATAGGAG	LR-PCR	chr3	128923007	128933995	10989
LR_422641_2	TTGCCCTGAGCTGTGAGAGTGACAACC	LR-PCR				
LR_422643_1	CATCACCGTGTACCAGGAAAACATTACTCA	LR-PCR	chr3	129005300	129009217	3918
LR_422643_2	AGTTCCTGCCCCTGTTCTTGTATGACTATGTC	LR-PCR				
LR_422644_1	ACTTCATTTTATGATGTTTTCTGACCCCTACT	LR-PCR	chr3	129009222	129019895	10674
LR_422644_2	CCCTTCTGAAAACAACAACAACAATAACAA	LR-PCR				
LR_422645_1	CCCATAGGCTCTCAAAATATGTTACTGTTATC	LR-PCR	chr3	128984139	128994959	10821
LR_422645_2	GCAGCATTTAGATTCCAAACTATCCAAT	LR-PCR				
LR_422646_1	AGCAGCAGTAGAAGTTGGTGGGTATCGC	LR-PCR	chr3	128973242	128984269	11028
LR_422646_2	CACCACTGGCTCTGCATCATTCTAGGACTTAG	LR-PCR				
LR_66676_1	AGACCGAAGAGAAGGACCACCATCAATAGAAT	LR-PCR	chr1	46637285	46644222	6938
LR_66676_2	TGAACACTCACCGCTTTGGAAACTAGGAC	LR-PCR				
LR_66677_1	GTGGCCATTTCCTGTTTCCAGCATCTTATGTT	LR-PCR	chr1	46644255	46653043	8789
LR_66677_2	CACGAGTCTAGTCCTCAGGCCGAGCTATGA	LR-PCR				
LR_724563_1	GGAACCTATAGAACCCTGGTGATCCTGTTACT	LR-PCR	chr3	128933578	128937991	4414
LR_724563_2	AGCCATTTCTCACGTGTTCTCTCTAGGAC	LR-PCR				
LR_724564_1	TCTCTCTGCTATGCGTGACCCTGGCTAACATC	LR-PCR	chr3	128960855	128963983	3129
LR_724564_2	GTAGGGTCAGGGCTGGCTTCGCTTTGA	LR-PCR				
LR_724565_1	GTGTCAAAGCGAAGCCAGCCCTGACCCTAC	LR-PCR	chr3	128963954	128973391	9438
LR_724565_2	CATGCTGCCCCTGGTGTTCTCAACATACGCTA	LR-PCR				
Adpt1.1	CTGAGATCGGAAGAGCTCGTATGCCGTCTTCTGCTTG	indexing-CAGT				
Adpt1.2	ACACTCTTTCCCTACACGACGCTCTTCCGATCTCAGT					
Adpt10.1	TAGAGATCGGAAGAGCTCGTATGCCGTCTTCTGCTTG	indexing-CTAT				
Adpt10.2	ACACTCTTTCCCTACACGACGCTCTTCCGATCTCTAT					
Adpt11.1	GCGAGATCGGAAGAGCTCGTATGCCGTCTTCTGCTTG	indexing-CGCT				
Adpt11.2	ACACTCTTTCCCTACACGACGCTCTTCCGATCTCGCT					
Adpt12.1	CCGAGATCGGAAGAGCTCGTATGCCGTCTTCTGCTTG	indexing-CGGT				
Adpt12.2	ACACTCTTTCCCTACACGACGCTCTTCCGATCTCGGT					
Adpt13.1	ACGAGATCGGAAGAGCTCGTATGCCGTCTTCTGCTTG	indexing-CGTT				
Adpt13.2	ACACTCTTTCCCTACACGACGCTCTTCCGATCTCGTT					
Adpt14.1	TGGAGATCGGAAGAGCTCGTATGCCGTCTTCTGCTTG	indexing-CCAT				
Adpt14.2	ACACTCTTTCCCTACACGACGCTCTTCCGATCTCCAT					
Adpt15.1	CGGAGATCGGAAGAGCTCGTATGCCGTCTTCTGCTTG	indexing-CCGT				
Adpt15.2	ACACTCTTTCCCTACACGACGCTCTTCCGATCTCCGT					
Adpt16.1	AGGAGATCGGAAGAGCTCGTATGCCGTCTTCTGCTTG	indexing-CCTT				
Adpt16.2	ACACTCTTTCCCTACACGACGCTCTTCCGATCTCCTT					
Adpt2.1	GAGAGATCGGAAGAGCTCGTATGCCGTCTTCTGCTTG	indexing-CTCT				
Adpt2.2	ACACTCTTTCCCTACACGACGCTCTTCCGATCTCTCT					
Adpt3.1	TCGAGATCGGAAGAGCTCGTATGCCGTCTTCTGCTTG	indexing-CGAT				
Adpt3.2	ACACTCTTTCCCTACACGACGCTCTTCCGATCTCGAT					
Adpt4.1	TTGAGATCGGAAGAGCTCGTATGCCGTCTTCTGCTTG	indexing-CAAT				
Adpt4.2	ACACTCTTTCCCTACACGACGCTCTTCCGATCTCAAT					
Adpt5.1	GGGAGATCGGAAGAGCTCGTATGCCGTCTTCTGCTTG	indexing-CCCT				
Adpt5.2	ACACTCTTTCCCTACACGACGCTCTTCCGATCTCCCT					
Adpt6.1	GTGAGATCGGAAGAGCTCGTATGCCGTCTTCTGCTTG	indexing-CACT				
Adpt6.2	ACACTCTTTCCCTACACGACGCTCTTCCGATCTCACT					
Adpt7.1	ATGAGATCGGAAGAGCTCGTATGCCGTCTTCTGCTTG	indexing-CATT				
Adpt7.2	ACACTCTTTCCCTACACGACGCTCTTCCGATCTCATT					
Adpt8.1	AAGAGATCGGAAGAGCTCGTATGCCGTCTTCTGCTTG	indexing-CTTT				
Adpt8.2	ACACTCTTTCCCTACACGACGCTCTTCCGATCTCTTT					
Adpt9.1	CAGAGATCGGAAGAGCTCGTATGCCGTCTTCTGCTTG	indexing-CTGT				
Adpt9.2	ACACTCTTTCCCTACACGACGCTCTTCCGATCTCTGT					
Solexa Pirmer1	AATGATACGGCGACCACCGAGATCTACACTCTTTCCC TACACGACGCTCT TCCGATCT	Library Enrichment				
Solexa Primer2	CAAGCAGAAGACGGCATACGAGCTCTTCCGATCT					

*Failed PCR amplification

Supplemental Table 7 : List of SNPs genotyped on MassARRAY platform for quality control

rsID	Chr	Coordinate (NCBI36)	MAF_CEU (HapMap)	Gene_symbol
rs4141964	1	46637627	0.417	FAAH
rs324420	1	46643348	0.225	
rs324419	1	46644573	0.178	
rs3773155	3	128921218	0.125	MGLL
rs3773159	3	128921628	0.142	
rs594323	3	128931676	0.425	
rs9759081	3	128942444	0.263	
rs497897	3	128952085	0.054	
rs17203659	3	128952276	0.224	
rs6778770	3	128956178	0.255	
rs17282181	3	128957231	0.225	
rs7652615	3	128963163	0.125	
rs9852837	3	128973744	0.051	
rs13066225	3	128974021	0.217	
rs567384	3	128976554	0.142	
rs13076593	3	128981216	0.158	
rs936839	3	128981668	0.051	
rs17203666	3	128985993	0.2955	
rs11715363	3	128998674	0.258	
