# Supplementary figures and images for: Population sequencing of two endocannabinoid metabolic genes identifies rare and common regulatory variants associated with extreme obesity and metabolite level
Source: Genome Biol. 2010 Nov 30;11(11):R118. doi: 10.1186/gb-2010-11-11-r118 (PMC3156957; doi:10.1186/gb-2010-11-11-r118)

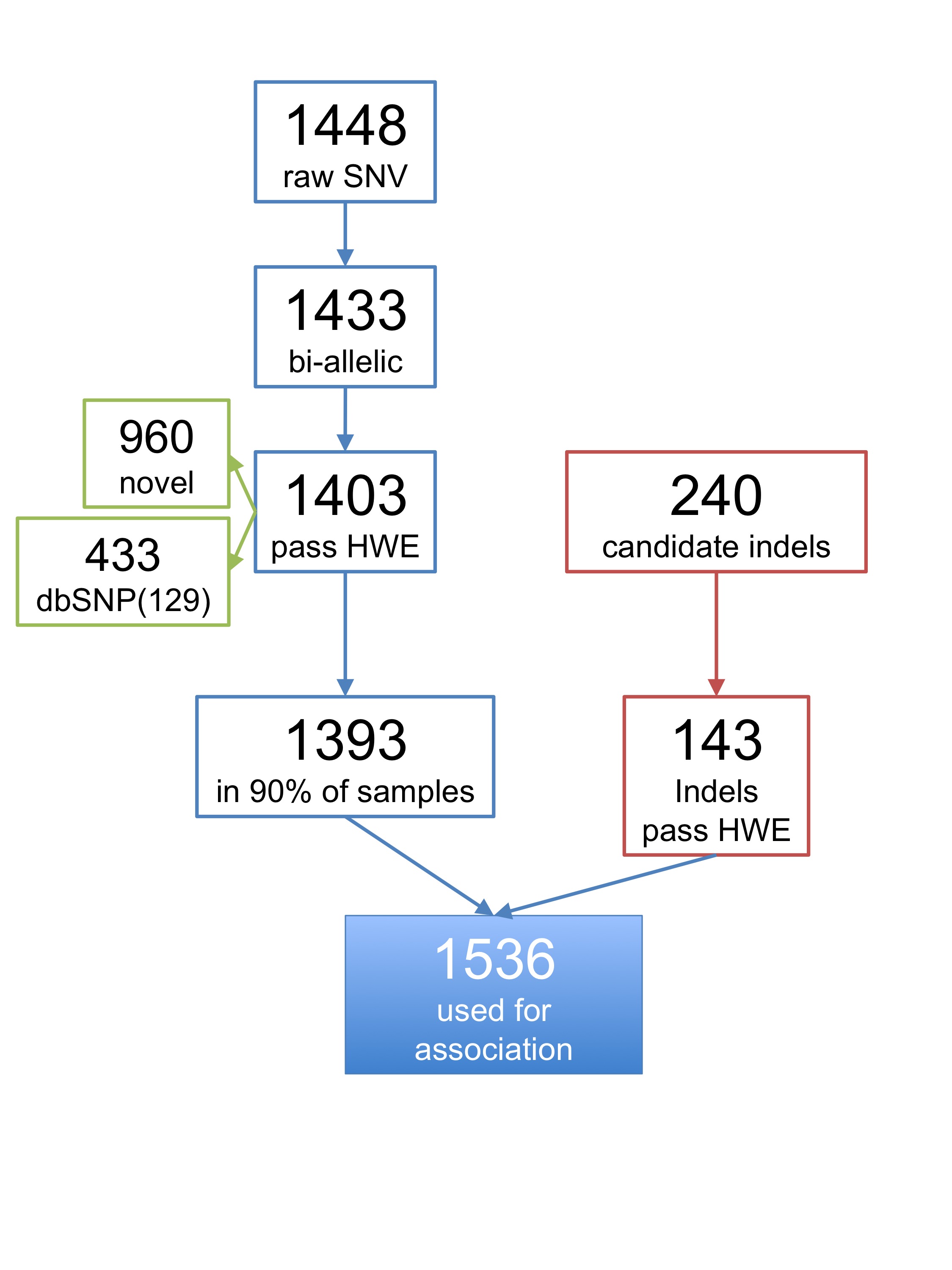

Supplement: Additional file 2 — Supplementary Figure S3. Flowchart illustrating the filtering steps for the variant calling. [file gb-2010-11-11-r118-S2.JPEG]

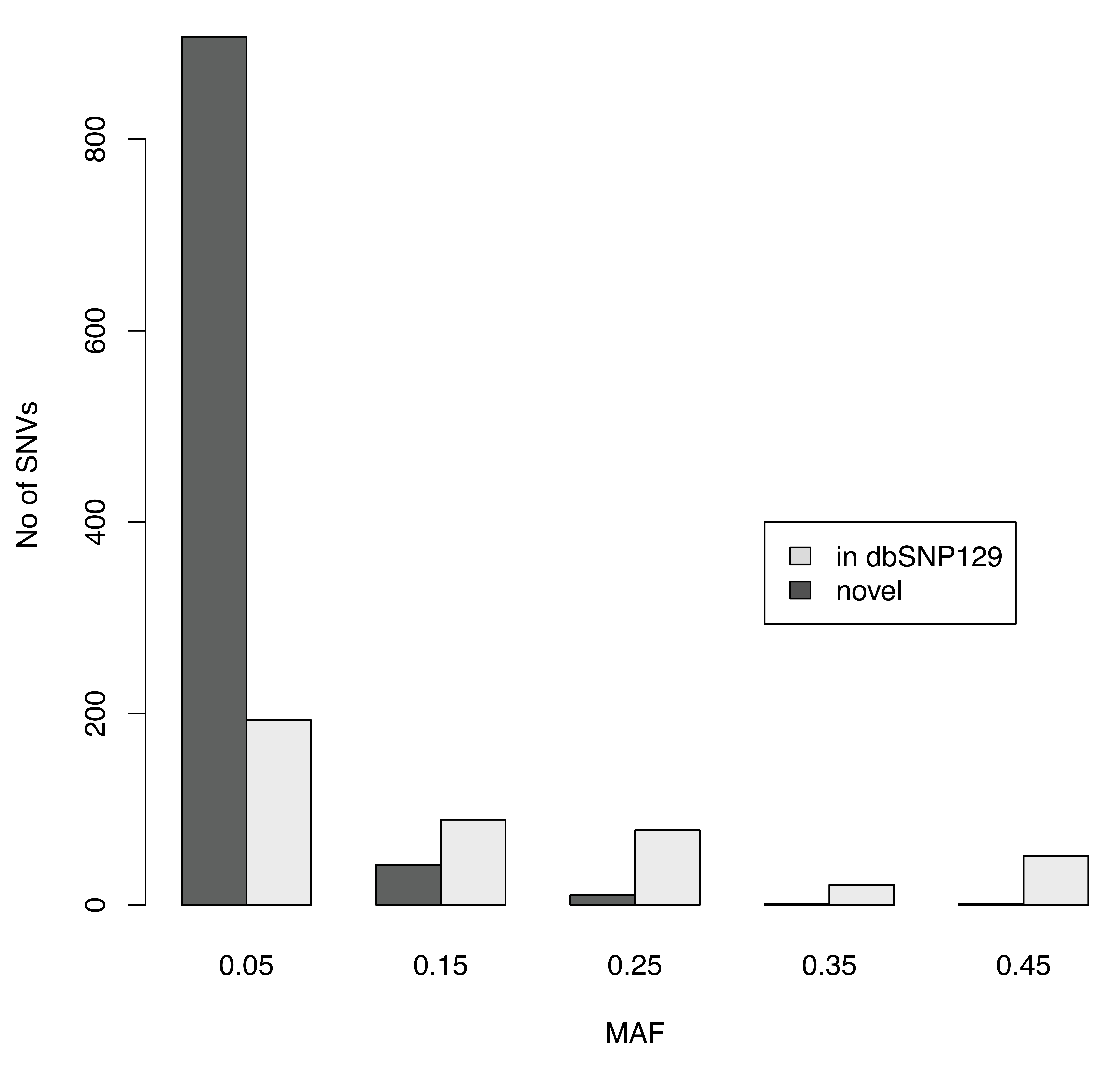

Supplement: Additional file 3 — Supplementary Figure S1. Distribution of the minor allele frequencies in the sequenced population for SNVs present in dbSNP (light grey) or novel SNVs (dark grey) in the FAAH and MGLL sequenced intervals. [file gb-2010-11-11-r118-S3.JPEG]

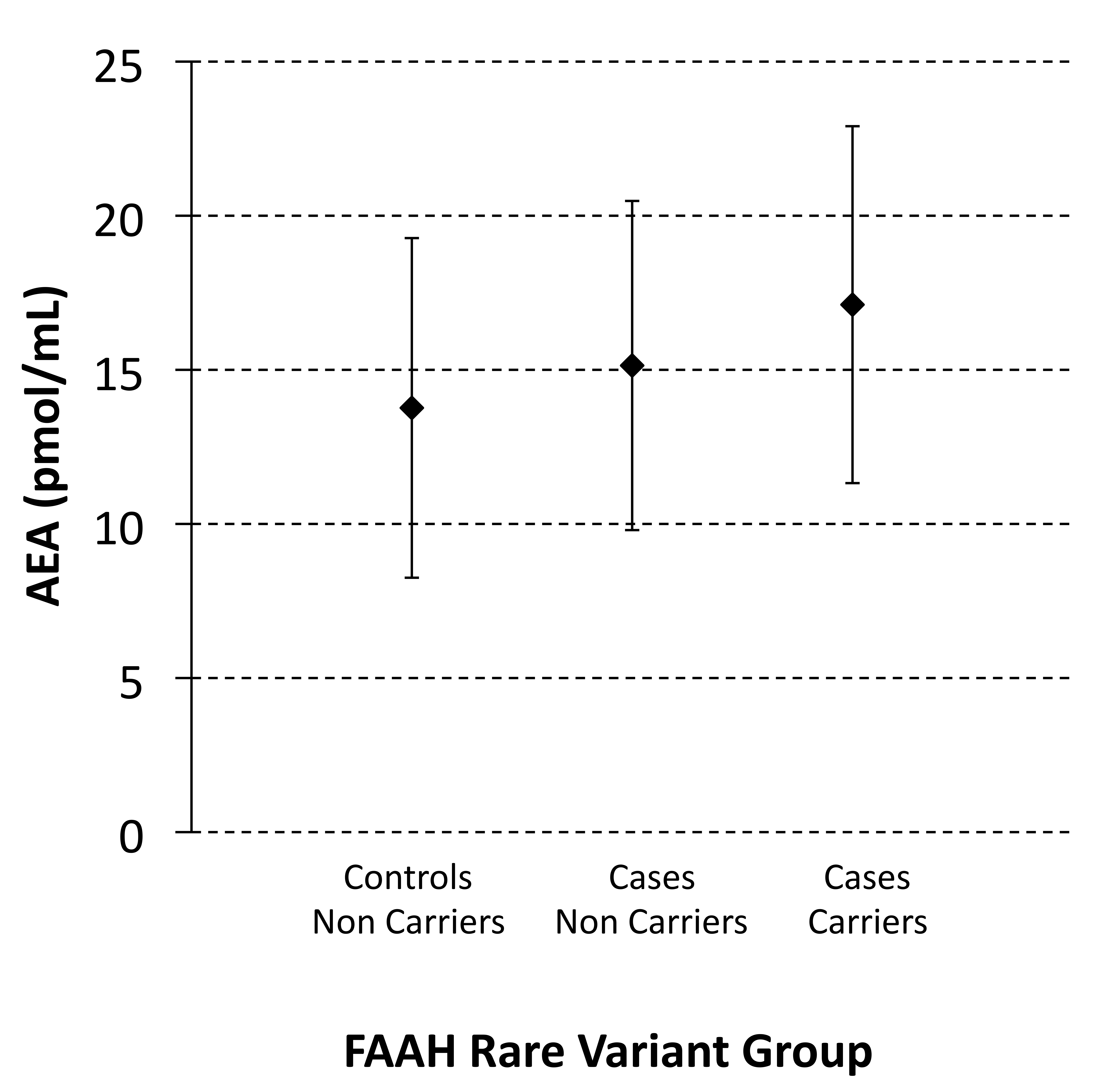

Supplement: Additional file 4 — Supplementary Figure S2. Average AEA plasma levels (pmol/ml) in 48 non-carriers controls, 80 non-carrier cases and 14 case carriers of the most significant FAAH variant-locus allele associated with high BMI. Error bars represent the standard deviation from the mean. [file gb-2010-11-11-r118-S4.JPEG]
